# Hospital Readmissions by Variation in Engagement in the Health Care Hotspotting Trial: A Secondary Analysis of a Randomized Clinical Trial

**DOI:** 10.1001/jamanetworkopen.2023.32715

**Published:** 2023-09-12

**Authors:** Qiang Yang, Dawn Wiest, Anna C. Davis, Aaron Truchil, John L. Adams

**Affiliations:** 1 Camden Coalition, Camden, New Jersey; 2 Center for Effectiveness and Safety Research, Kaiser Permanente, Pasadena, California; 3 Department of Health Systems Science, Kaiser Permanente Bernard J. Tyson School of Medicine, Pasadena, California

## Abstract

**Importance:**

Variability in intervention participation within care management programs can complicate standard analysis strategies.

**Objective:**

To evaluate whether care management was associated with reduced hospital readmissions among individuals with higher participation probabilities.

**Design, Setting, and Participants:**

A total of 800 hospitalized patients aged 18 years and older were randomized as part of the Health Care Hotspotting randomized clinical trial, which was conducted in Camden, New Jersey, from June 2014 to September 2017. Data were collected through October 2018. In this new analysis performed between April 6, 2022, and April 23, 2023, the distillation method was applied to account for variable intervention participation. A gradient-boosting machine learning model produced predicted probabilities of engaged participation using baseline covariates only. Predicted probabilities were used to trim both intervention and control populations in an equivalent manner, and intervention effects were reevaluated within study population subsets that were increasingly concentrated with patients having higher participation probabilities. Patients had 2 or more hospitalizations in the 6-month preenrollment period and documented evidence of chronic illness and social complexity.

**Intervention:**

Multidisciplinary teams provided services to patients in the intervention arm for a mean 120 days after hospital discharge. Patients in the control group received usual postdischarge care.

**Main Outcomes and Measures:**

Hospital readmission rates and counts 30, 90, and 180 days postdischarge.

**Results:**

Of 800 eligible patients, 782 had complete discharge information and were included in this analysis (mean [SD] age, 56.6 [12.7] years; 395 [50.5%] female). In the intent-to-treat analysis, the unadjusted 180-day readmission rate for treatment and control groups was 60.1% vs 61.7% (adjusted odds ratio, 0.95; 95% CI, 0.71-1.28; *P* = .73) and the mean (SD) number of 180-day readmissions was 1.45 (1.89) vs 1.48 (1.94) (adjusted incidence rate ratio, 0.99, 95% CI, 0.88-1.12; *P* = .86). Among the population with the highest participation probabilities, the mean (SD) 180-day readmission count was 1.22 (1.74) vs 1.57 (1.74) and the incidence rate ratio attained statistical significance (adjusted incidence rate ratio, 0.74; 95% CI, 0.56-0.99; *P* = .045). Adjusted odds ratios and adjusted incidence rate ratios for 30- and 90-day outcomes reached statistical significance after population distillation.

**Conclusions and Relevance:**

This secondary analysis of a randomized clinical trial found that care management was associated with reduced readmissions among patients with higher participation probabilities, suggesting that program operation could be improved by addressing barriers to participation and refining inclusion criteria to identify patients most likely to benefit.

**Trial Registration:**

ClinicalTrials.gov Identifier: NCT02090426

## Key Points

**Question:**

Were outcomes following the Health Care Hotspotting intervention different for those patients who were more vs less likely to engage with care management?

**Findings:**

In this secondary analysis of randomized clinical trial data from 782 participants, greater intervention participation was associated with significantly lower readmission rates 30 and 90 days after hospital discharge and significantly lower 30- and 180-day readmission counts.

**Meaning:**

These findings suggest that evaluation strategies that account for variable intervention participation can identify program effects that are missed in intent-to-treat analysis.

## Teaser

This secondary analysis of a randomized clinical trial evaluates the association between care management and hospital readmissions among trial participants with greater engagement.

## Introduction

Randomized evaluations of complex care management programs have yielded mixed evidence of effectiveness in improving health outcomes. An approach to care for individuals with concurrent medical and social needs, complex care is distinct from other care management approaches for its interdisciplinary team-based models, prioritization of patients’ self-defined goals, and emphasis on cross-sector partnerships to improve care transitions across multiple settings.^1,2^ These programs tend to focus on high-cost, high-needs populations,^3,4,5^ and their value is often framed as cost savings achieved through reductions in avoidable hospitalizations and emergency department use.^6,7^ Several randomized clinical trials have shown positive effects with complex care programs in reducing costs,^8,9^ hospitalizations,^8,10,11,12^ or emergency department visits,^10^ while others have produced null results for similar outcomes.^8,9,10,13,14,15,16^

The Camden Coalition received support in 2012 from the US Centers for Medicare and Medicaid Services to refine, test, and scale their signature care management model: the Camden Core Model. In 2020, randomized clinical trial results challenged a key value proposition for complex care, finding no significant effect of the Core Model on readmissions 180 days after hospital discharge.^16^

The Health Care Hotspotting trial randomized patients after recruitment and consent, leading to complete trial enrollment within the intervention group. However, patients enrolled in the trial varied on characteristics that could have influenced participation in and responsiveness to the intervention, and while enrollment was complete within the intervention group by virtue of the trial design, the intensity and nature of care services received by enrollees varied.^17^

The original analysis of the Health Care Hotspotting trial adhered to the standard intent-to-treat (ITT) framework, in which patients are analyzed as randomized. However, variable intervention delivery meant that the average treatment effect in the main analysis was a blended estimate of effectiveness across varying doses of intervention services.

In this new analysis of Health Care Hotspotting trial data, we applied the distillation method, a framework for analyzing data from randomized clinical trials that are underpowered due to low participant enrollment or variable participation levels while maintaining the trial’s original randomization.^18^ We used data on intervention participation to develop a model for estimating engagement and assessed intervention effectiveness in narrower subsets of the study population consisting of participants from both trial groups with higher probabilities of intervention engagement. The revised eligibility criteria, based on prerandomization variables, were applied to both the treatment and control groups.

## Methods

### Study Setting

This was a secondary analysis of a randomized clinical trial of the Camden Core Model. From June 2014 to September 2017, 800 hospitalized patients were randomized using a tamper-proof and externally recorded randomization device to intervention vs usual care. The primary prespecified outcome was readmission within 180 days after hospital discharge. The protocol was approved by Cooper University Healthcare, the National Bureau of Economic Research, and Our Lady of Lourdes Medical Center, and written informed consent was obtained from all participants. We used the Consolidated Standards of Reporting Trials (CONSORT) checklist when writing this report.^19^

**Figure 1.**
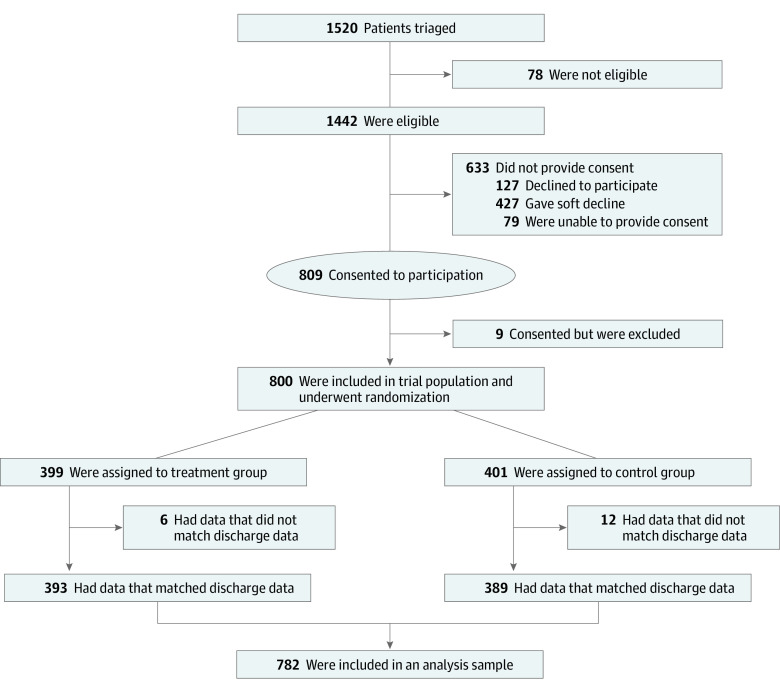
Flow Diagram Patients who declined to participate explicitly said no to the offer of randomization. Patients who gave a soft decline did not provide consent when approached but did not decline to participate and could be approached again during future hospitalizations if they were otherwise eligible. Patients who were unable to provide consent were either discharged or died before they could be reached or were unable to consent for reasons such as being asleep. Patients who consented but were excluded included 5 patients who consented and later asked to be removed from the trial and the last 4 patients enrolled in the trial who were excluded to keep the trial population at the target of 800 patients. For patients in the trial population to be included in the analysis sample, a record of their index admission had to have been found in the hospital discharge data. Further information is provided in Supplement 1.

Patients were identified through a Health Information Exchange, and study recruitment took place at 2 hospitals in Camden, New Jersey. Eligible patients were 18 years and older with 2 or more hospitalizations in the prior 6 months with evidence of multiple chronic illnesses and social complexity in their medical records. Care staff met eligible patients at hospital bedside to invite them to participate in the study and generated the random allocation sequence. Consented patients were randomized into intervention and control arms, with 399 patients enrolled in the treatment group to receive care management services and 401 randomized into the control group to receive usual postdischarge care. The final analytic sample consisted of 782 patients (393 in the treatment group and 389 in the control group) with complete discharge information. Outcome data were collected through October 2018. The participant flow diagram is shown in Figure 1; the full trial protocol is included in Supplement 1.

Patients enrolled in the intervention arm of the trial received care management services from registered nurses, licensed practical nurses, social workers, health coaches, and community health workers for a mean 120 days after discharge from their index admission. The details of the intervention are described in the original trial report.^16^

### Data Sources

Data sources for the study included all-payer claims data from 5 regional hospital systems, a care coordination database, and information collected from all study participants during triage and the initial hospital visit. Claims data covered 6 months prior to and 6 months after each patient’s index discharge. These data were at the hospital-encounter level and had admission and discharge dates along with *International Classification of Diseases, Ninth Revision *(*ICD-9*) and *Tenth Revision* (*ICD10*) diagnostic codes. Care coordination records held information from in-person meetings and phone calls with patients as well as clinical and social need coordination activities carried out by staff. Before randomization, care staff collected demographic, clinical, and social information from both intervention and control group patients as part of their preenrollment bedside engagement. Race and ethnicity were reported by patients in response to questions on the preenrollment survey administered by care team members in order to assess the generalizability of the results. Additional details about all data sources can be found in eAppendix 1 in Supplement 2.

### Statistical Analysis

The original analysis of trial data was completed using ITT estimates and has been published elsewhere.^16^ Given the variability in intervention intensity delivered to patients in the intervention arm (eFigure in Supplement 2), we undertook this reanalysis of trial data to assess intervention effectiveness when heterogeneity in treatment intensity is addressed. The statistical approach used for this new analysis leverages a strategy that accounts for low participant responsiveness in the intervention arm while retaining the benefits of the original randomization.^18^ This method permits an assessment of intervention effectiveness when trial eligibility criteria are narrowed to the population that was more likely to receive an adequate intervention dose. All analyses were performed between April 6, 2022, and April 23, 2023, using RStudio version 2022.02.0 (R Foundation). The R extended packages used were dplyr for data processing, gbm for gradient-boosting machine modeling, Surv for survival analysis, and ggplot2 for data visualizations. All tests unless specified otherwise are 2-tailed tests with significance level at α = .05.

#### Stage 1: Estimating Participation

##### Participation Definition

An ITT framework would consider all patients randomized to the intervention group as participants. We developed a participation definition for the purposes of reanalysis that acknowledged varied participation levels by categorizing intervention group enrollees as engaged participants or nonparticipants. Engaged participants were individuals in the treatment arm who met at least 2 of the 3 following criteria: received at least 3 intervention hours during the first 2 weeks of enrollment, had contact with staff at least once per week for 4 of the initial 6 weeks, were retained in the program for 60 days (half of the average treatment length) or graduated within that timeframe.

The thresholds were chosen based on each measure’s distribution, together with the logical design of the intervention. The beginning period of the intervention was considered crucial as staff collected information, conducted home visits, designed care plans with patients, and built relationships, and the intervention was designed in a continuous way such that patients were expected to be engaged at least once weekly. Results of sensitivity analyses using different thresholds for the measures of engaged participation are shown in eAppendix 2 in Supplement 2.

##### Independent Variables and Outcome Variable

In stage 1 of the distillation analysis, a machine learning approach was used to develop predicted probabilities of engaged participation in the intervention based on prerandomization covariates. The assumption for the stage 1 model is that estimations from this model will associate with the person-level treatment effects. Failing this assumption, the method will not increase power to detect treatment effects. Patients’ demographic, social, clinical, and health utilization variables for only the baseline period were included in stage 1 modeling to estimate engaged participation. All variables were available for both intervention and control group patients. The complete list of variables is included in eAppendix 3 in Supplement 2. The outcome variable in stage 1 modeling was the engaged participation indicator within the intervention group, based on the criteria described previously.

##### Modeling

We applied a gradient-boosting machine model to estimate engaged participation within the intervention group. The gradient-boosting machine model optimized the area under the receiver operating characteristic curve using 2 -way interactions of the independent variables and a learning rate of 0.001.^20^ Eight-fold cross-validation was used to select the optimization parameters and avoid overfitting.^20^ The model was allowed to self-select up to 10 000 trees, the exact number depending on the cross-validation. This combination of cross-validation, low learning rate, relatively low depth of interaction, and a reasonably high number of trees allows complexity of associations among the independent variables to be captured by the model with a controlled risk of over-fitting.

#### Stage 2: Population Distillation

The stage 1 model was built with intervention patients only and then used to estimate the probability of engaged participation for all patients in the study population. The estimations from the model are a fixed function of prerandomization covariates, which were applied to both treatment and control groups equally. In the control population, these represent the probability that patients would have engaged had they been randomized to the treatment arm. The ordered probabilities were then divided into deciles, allowing us to narrow the full study population to subsets that were increasingly enriched with likely participants. We defined these distilled subsets based on the top percentage most likely to be engaged participants, ranging from 100% (the full population) to 20% (the 20% most likely to be engaged participants).

#### Stage 3: Estimating Readmission Outcomes

In the third stage of the reanalysis, we fit regression models to decreasing fractions of the study population representing the distilled subsets to assess differences in outcomes between intervention and control group patients as the sample was sequentially concentrated by excluding patients from both trial arms with lower probabilities of engaged participation. We studied readmission rates and the average number of readmissions at 30, 90, and 180 days after patients’ index discharge dates controlling for key covariates (eAppendix 3 in Supplement 2). We applied multivariable Poisson regression models to readmission counts and multivariable logistic regression models to readmission rates. After distillation, the resulting analysis is a simple generalized linear model regression analysis with binomial or Poisson distribution assumptions. Stage 3 models were run with only a treatment group indicator (unadjusted) and with relevant covariates (adjusted) to account for potential group differences despite randomization. Formats of the covariates and criteria for their inclusion in the final model are described in eAppendix 3 in Supplement 2; Poisson model validations are presented in eAppendix 4 in Supplement 2.

## Results

### Estimating Intervention Exposure or Dosage

In total, 782 patients were included in analysis (mean [SD] age, 56.6 [12.7] years; 395 [50.5%] female and 387 [49.5%] male; 231 [29.5%] Hispanic, 427 [54.6%] non-Hispanic Black, and 124 [15.9%] non-Hispanic White). Our gradient-boosting machine model estimating intervention exposure performed well with an area under the receiver operating characteristic curve (SD) of 0.81 (0.02). Factors with the greatest associations were patient age, prior number of hospitalizations, length of index admission, housing status at enrollment, and arrest history (eAppendix 3 in Supplement 2).

### Population Distillation: Change in Characteristics

As the population became more concentrated among those with the highest probabilities of engaged participation, a larger proportion of the most distilled sample were female (50.5% to 60.9%), were of Hispanic ethnicity (29.5% to 52.6%), did not have a high school degree (46.4% to 76.9%), reported having sufficient family support (59.8% to 69.9%), and had higher occurrences of specific chronic illnesses, including chronic obstructive pulmonary disease (44.5% to 67.9%) and kidney disease (31.6% to 57.9%). Patients with the highest probabilities of engaged participation were less likely to have been arrested (6.0% to 0.0%), to have had a substance use (32.6% to 25.6%) or alcohol-specific (13.4% to 7.1%) diagnoses at the hospital, and to have mild liver disease (11.9% to 2.6%) or moderate to severe liver disease (4.0% to 0.6%) (Table 1). The breakdown for treatment and control groups in the percentage change in characteristics as the population became more concentrated is presented in eTable in Supplement 2.

**Table 1.** Patient Characteristics Within Increasingly Distilled Samples

Characteristic	Percentage of population selected, No. (%)		
	100% (N = 782)	60% (n = 469)	20% (n = 156)
Age, mean (SD), y	56.6 (12.7)	59.9 (9.3)	62.0 (6.7)
Sex			
Male	387 (49.5)	217 (46.3)	61 (39.1)
Female	395 (50.5)	252 (53.7)	95 (60.9)
Race and ethnicity			
Hispanic^a^	231 (29.5)	170 (36.2)	82 (52.6)
Non-Hispanic Black^a^	427 (54.6)	254 (54.2)	70 (44.9)
Non-Hispanic White^a^	124 (15.9)	45 (9.6)	4 (2.6)
Education			
≥High school degree	419 (53.6)	215 (45.8)	36 (23.1)
No high school degree	363 (46.4)	254 (54.2)	120 (76.9)
Relationship status			
Married/partnered	186 (23.8)	121 (25.8)	39 (25.0)
Single/divorced/widowed	596 (76.2)	348 (74.2)	117 (75.0)
Housing			
Stably housed	705 (90.2)	442 (94.2)	150 (96.2)
Experiencing homelessness	77 (9.8)	27 (5.8)	6 (3.8)
Family support			
Sufficient family support	468 (59.8)	295 (62.9)	109 (69.9)
Insufficient family support	314 (40.2)	147 (37.1)	47 (30.1)
Employment			
Employed	43 (5.5)	5 (1.1)	0 (0.0)
Not employed	739 (94.5)	464 (98.9)	156 (100.0)
Any arrest in prior 6 mo	47 (6.0)	0 (0.0)	0 (0.0)
Self-reported health			
Fair/good/excellent	363 (46.4)	204 (43.5)	67 (42.9)
Poor	419 (53.6)	265 (56.5)	89 (57.1)
No. admissions in prior 6 mo, mean (SD)	2.7 (1.6)	2.4 (1.0)	2.0 (0.4)
Index admission, No. of days, mean (SD)	7.0 (5.8)	6.6 (4.3)	6.2 (3.4)
Alcohol use diagnosis	105 (13.4)	46 (9.8)	11 (7.1)
Mental health diagnosis	520 (66.5)	309 (65.9)	107 (68.6)
Substance use diagnosis	255 (32.6)	124 (26.4)	40 (25.6)
AIDS	16 (2.0)	8 (1.7)	1 (0.6)
Chronic obstructive pulmonary disease	348 (44.5)	249 (53.1)	106 (67.9)
Congestive heart failure	278 (35.5)	175 (37.3)	60 (38.5)
Dementia	8 (1.0)	6 (1.3)	1 (0.6)
Diabetes with complication	174 (22.3)	135 (28.8)	62 (39.7)
Hemiplegia or paraplegia	15 (1.9)	8 (1.7)	1 (0.6)
Mild liver disease	93 (11.9)	42 (9.0)	4 (2.6)
Moderate or severe liver disease	31 (4.0)	16 (3.4)	1 (0.6)
Kidney disease	247 (31.6)	186 (39.7)	81 (51.9)
Rheumatoid arthritis	31 (4.0)	19 (4.1)	6 (3.8)
Anxiety disorder	175 (22.4)	93 (19.8)	36 (23.1)
Mood disorder	239 (30.6)	133 (28.4)	56 (35.9)
Schizophrenia	48 (6.1)	29 (6.2)	10 (6.4)
Suicidal ideation	32 (4.1)	14 (3.0)	3 (1.9)

^a^ Four patients indicated another race on the baseline survey (Asian, multiracial, or other). Because of modeling requirements, categories with only 4 elements could not be included in analysis. We therefore assigned these patients the most probable race and ethnicity category (Hispanic, non-Hispanic Black, or non-Hispanic White) such that the assigned category of each patient had the largest probability of association with their engaged participation outcome label.

### Evaluating Intervention Effectiveness

**Figure 2.**
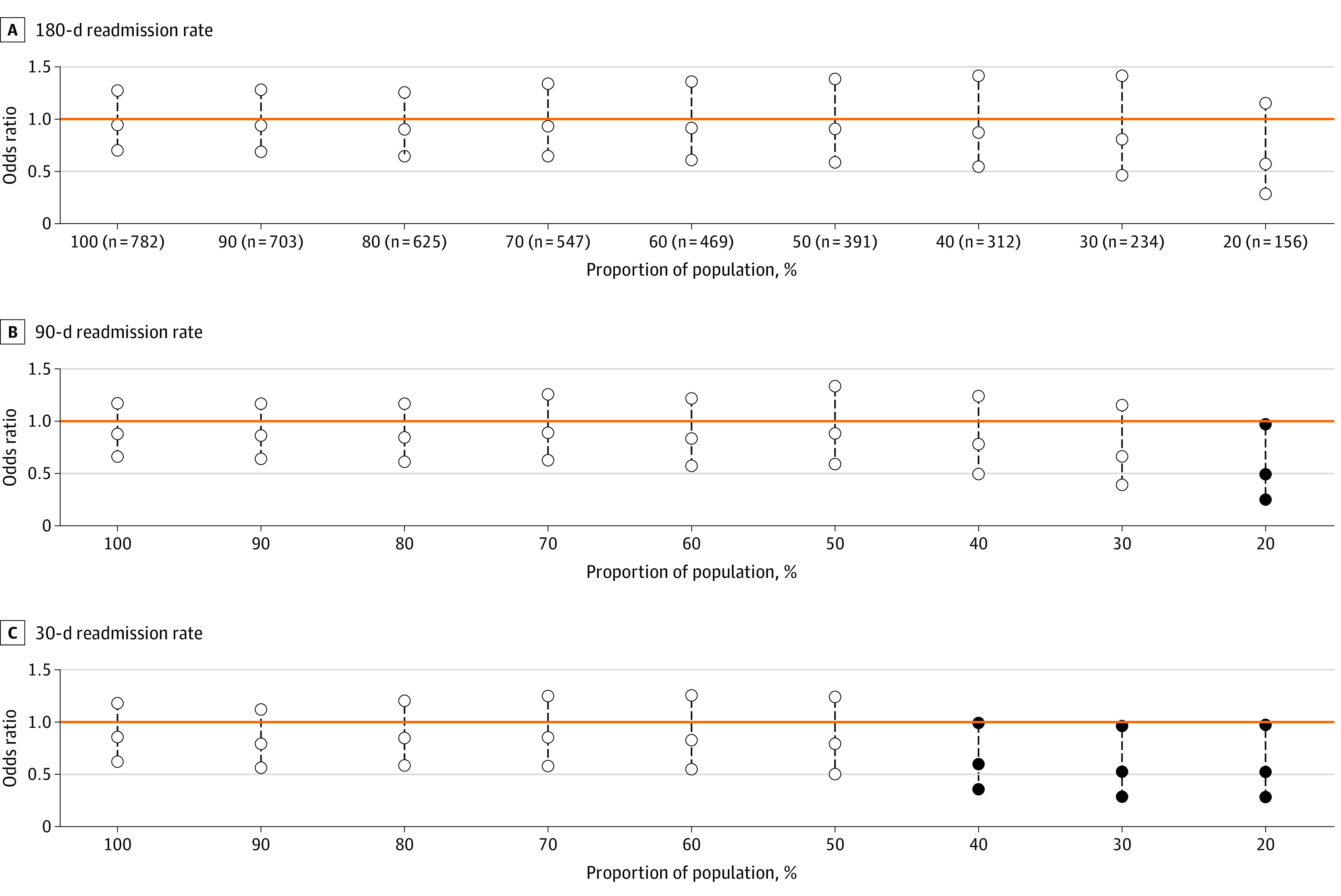
Readmission Rates Between Intervention and Control Group Patients Within Increasingly Distilled Samples Odds ratios and 95% CIs shown. In each panel, a CI is plotted at each percentage of population distillation with the center circle representing the estimated value and the outer circles representing the end points of the interval. If the interval is completely below or above the dotted reference line (y = 1), a solid circle indicates statistical significance.

**Figure 3.**
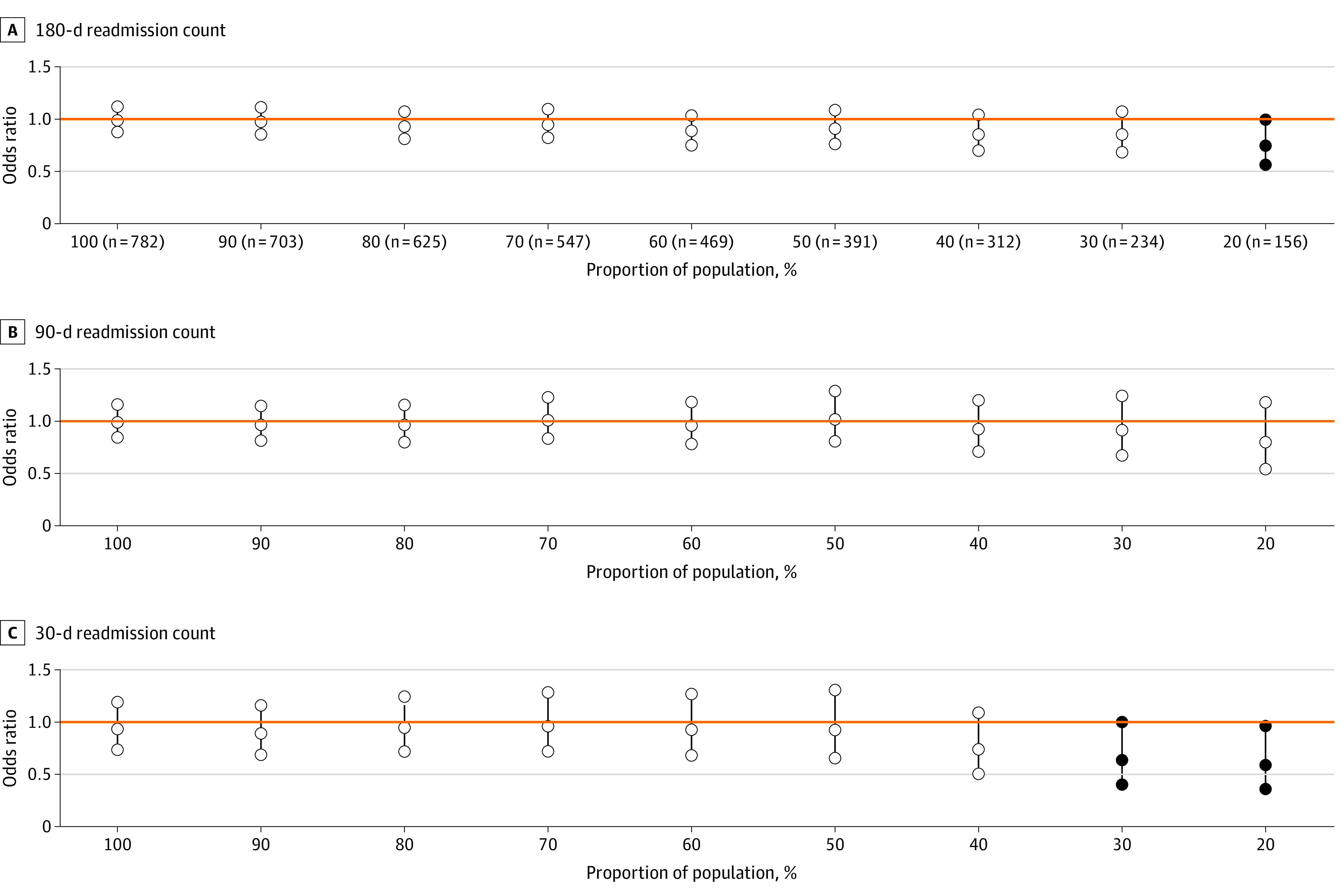
Readmission Counts Between Intervention and Control Group Patients Within Increasingly Distilled Samples Incidence rate ratios and 95% CIs shown. In each panel, a CI is plotted at each percentage of population distillation with the center circle representing the estimated value and the outer circles representing the end points of the interval. If the interval is completely below or above the dotted reference line (y = 1), a solid circle indicates statistical significance.

In the odds ratio formulation for readmission rates (Figure 2), after 50%, we observed a consistent downward trend in the point estimates representing greater intervention effectiveness as distillation increased. A similar pattern was found in the incidence rate ratio formulation for readmission counts (Figure 3), with a less pronounced downward trend. The patterns indicate that the intervention was associated with decreased readmissions beginning at a moderate level of distillation.

Table 2 shows unadjusted and adjusted readmission results for the intervention and control groups at different distillation levels. In unadjusted analysis, the 180-day readmission rate for treatment and control groups was 60.1% vs 61.7%. In adjusted analysis, the relative readmission risk between intervention and control group patients declined from 0.95 (95% CI, 0.71-1.28; *P* = .73) for the full population to 0.57 (95% CI, 0.28-1.15; *P* = .12) when the population was narrowed to the 20% most likely to engage. For the 180-day readmission count, the mean (SD) number of 180-day readmissions was 1.45 (1.89) vs 1.48 (1.94) (adjusted incidence rate ratio, 0.99; 95% CI, 0.88-1.12; *P* = .86). Among the population with the highest participation probabilities, the mean (SD) 180-day readmission count was 1.22 (1.74) vs 1.57 (1.74) and the adjusted incidence rate ratio attained statistical significance (0.74; 95% CI, 0.56-0.99; *P* = .045). For 30-day readmissions, the adjusted odds ratio was 0.85 (95% CI, 0.62-1.18; *P* = .34) for the full population and decreased to 0.52 (95% CI, 0.28-0.97; *P* = .04) when the population was narrowed to 20% population distillation. A similar trend was observed for 30-day readmission counts, with an incidence rate ratio of 0.94 (95% CI, 0.73-1.19; *P* = .59) for the full population and 0.59 (95% CI, 0.36-0.96; *P* = .03) at 20% population distillation. For the 90-day readmission outcomes, the rate formulation was significant at 20% of population distillation (OR, 0.48; 95% CI, 0.24-0.97; *P* = .04) but the count formulation was never significant (Figures 2 and 3 and Table 2). Results from additional analyses using alternative methods are like those reported here (eAppendix 5 in Supplement 2).

**Table 2.** Readmission Outcomes With Differences Between Control and Treatment Groups Within Increasingly Distilled Samples

Outcome by percentage of population^a^	Control	Treatment	Unadjusted measure (95% CI)^b^	Adjusted measure (95% CI)
**Rate, %**				
30 d				
100	30.1	26.7	OR, 0.85 (0.68-1.05)	OR, 0.85 (0.62-1.18)
60	31.1	26.6	OR, 0.80 (0.54-1.20)	OR, 0.82 (0.54-1.25)
20	31.2	19.0	OR, 0.52 (0.25-1.09)	OR, 0.52 (0.28-0.97)
90 d				
100	50.6	47.1	OR, 0.87 (0.65-1.15)	OR, 0.87 (0.65-1.17)
60	51.6	47.5	OR, 0.85 (0.59-1.22)	OR, 0.83 (0.57-1.22)
20	51.9	38.0	OR, 0.57 (0.30-1.07)	OR, 0.48 (0.24-0.97)
180 d				
100	61.7	60.1	OR, 0.93 (0.70-1.24)	OR, 0.95 (0.71-1.28)
60	64.9	62.6	OR, 0.91 (0.62-1.33)	OR, 0.92 (0.62-1.36)
20	66.2	53.2	OR, 0.58 (0.30-1.10)	OR, 0.57 (0.28-1.15)
**Count, mean**				
30 d				
100	0.38	0.35	IRR, 0.93 (0.84-1.02)	IRR, 0.94 (0.73-1.19)
60	0.39	0.36	IRR, 0.91 (0.80-IRR, 1.03)	IRR, 0.93 (0.68-1.27)
20	0.42	0.24	IRR, 0.58 (0.47-0.71)	IRR, 0.59 (0.36-0.96)
90 d				
100	0.89	0.88	IRR, 0.99 (0.82-1.18)	IRR, 0.99 (0.84-1.16)
60	0.88	0.84	IRR, 0.95 (0.76-1.18)	IRR, 0.96 (0.78-1.18)
20	0.84	0.72	IRR, 0.85 (0.59-1.24)	IRR, 0.80 (0.54-1.18)
180 d				
100	1.48	1.45	IRR, 0.98 (0.75-1.28)	IRR, 0.99 (0.88-1.12)
60	1.56	1.32	IRR, 0.84 (0.61-1.17)	IRR, 0.88 (0.75-1.03)
20	1.57	1.22	IRR, 0.77 (0.45-1.34)	IRR, 0.74 (0.56-0.99)

Abbreviations: IRR, incidence rate ratio; OR, odds ratio.

^a^ Sample sizes: 100% (N = 782); 60% (n = 469); 20% (n = 156).

^b^ Calculations of unadjusted ORs and IRRs are based on the regression models with only the treatment group indicator included. Calculations of the adjusted ORs and IRRs are based on the full regression models with the treatment indicator and other covariates (eAppendix 3 in Supplement 2). All 95% CIs were calculated with standard errors derived from the Fisher information matrix.

## Discussion

In this secondary analysis of a randomized clinical trial, when analysis of program outcomes was concentrated on the population most likely to engage with the intervention, the estimated effectiveness of the Camden Core Model intervention increased, and the confidence intervals became statistically significant. Like other randomized evaluations with variability in patient uptake,^10,21^ engagement,^22,23^ or intervention delivery,^24,25^ the Health Care Hotspotting trial would have benefitted from increased power to account for intervention exposure heterogeneity. When randomized evaluations cannot consider such heterogeneity in their power calculations, the distillation method complements the ITT framework by building upon the randomized design.^18^

The central motivation for the distillation method is that ITT methods for analyzing RCTs may lead to missed insights. Even when randomization occurs after patient outreach, the average treatment effect in ITT analysis is for the offer of the treatment rather than the delivery of the service itself. If a clinical trial has complete uptake and full treatment dose for all treatment arm participants, there is no difference between these concepts. However, as the rate of successful intervention delivery moves further from ideal, the ITT estimate may diverge from the intervention delivery effect. Beyond the power implications of this dilution, the ITT estimate may no longer estimate the effect that is of most policy or practical interest.

Randomized clinical trials of care management interventions can be prone to incomplete enrollment or engagement because these interventions are typically prolonged and multifaceted, unlike treatments that are assigned at a point in time (ie, insurance coverage) or given immediately after randomization (ie, a vaccine). Ultimately, ITT analyses of diluted trials may overlook the effect of a treatment on the treated, potentially mistaking trial implementation challenges for an intervention failure.

In our analysis, a history of criminal justice involvement and housing instability were associated with lower probabilities of engaged participation. These findings may reflect the difficulty of maintaining contact with participants whose lives are in flux, highlighting the importance of engagement strategies tailored to patient circumstances and needs.^26,27^ Care management programs could approach these findings in 2 ways: refine inclusion criteria to identify those most likely to engage and benefit or use engagement tools to better activate and support patients. Both approaches require building robust data capabilities to identify risk factors before and during initial participant enrollment.

One of the Camden Coalition’s key takeaways from the Health Care Hotspotting trial’s null results was that broader investments in social services and other resources are needed to improve outcomes for individuals living with complex health and social needs. During and after the initial study, the Camden Coalition expanded its services in response to patient needs, including developing Housing First and Medical Legal Partnership programs, and deepening partnerships with addiction medicine and behavioral health providers across the region. This new analysis emphasizes the value of those activities, given that patients who were experiencing homelessness and other social needs were among the least likely to fully engage in the intervention.

### Limitations

Our study has several limitations. The Camden Core Model did not establish a priori criteria to define full intervention exposure at the time of trial implementation. We used a combination of data distributions and guidance from program designers to select the engagement definition used for this analysis and tested several variations in sensitivity analyses. Although our study provided insight into the characteristics of patients who were less likely to be engaged, we were unable to assess causes of variable intervention exposure. Our results should not be interpreted to suggest that unengaged patients would have achieved outcomes comparable with engaged patients had they received a more complete intervention dose.

We analyzed readmissions at 180 days as the primary outcome for consistency with the original evaluation. However, the focus on this singular outcome measure as the primary value mechanism for complex care may distract from a broader range of potential benefits. While there are patients for whom reducing hospitalizations is an appropriate goal of complex care management, for others, frequent hospitalizations result from exceptional circumstances that are not resolved irrespective of intensive intervention.^16,28^ Other intervention outcomes such as care continuity and improved chronic disease management should be introduced into randomized evaluations along with patient-reported measures to advance a more holistic understanding of program value.^7,27,28,29^

## Conclusions

The conclusion from the original randomized clinical trial of the Camden Core Model that the intervention did not work may have been premature, because the estimated treatment effect in the ITT analysis was averaged over a population including patients who received low-intensity and short-term intervention. In this reanalysis of the Health Care Hotspotting trial, the distillation method helped identify individuals for whom intervention delivery was successful and whether the intervention worked when it could be delivered more fully. Both questions are essential to producing policy relevant results in evaluations of care management programs.

## Notes

Supplement 1.
Trial Protocol

Supplement 2. **eAppendix 1.** Data sources **eAppendix 2.** Sensitivity analysis of engaged participation definition **eAppendix 3.** Model building **eAppendix 4.** Poisson model validations **eAppendix 5.** Additional analysis **eTable.** Intervention and control group patient characteristics within increasingly distilled samples **eFigure.** Number of intervention hours received by treatment arm patients during their first week of intervention enrollment
eReferences

Supplement 3.
Data Sharing Statement
