# Supplementary material for: Hospital Readmissions by Variation in Engagement in the Health Care Hotspotting Trial: A Secondary Analysis of a Randomized Clinical Trial
Source: JAMA Netw Open. 2023 Sep 12;6(9):e2332715. doi: 10.1001/jamanetworkopen.2023.32715 (PMC10498327; doi:10.1001/jamanetworkopen.2023.32715)
Supplement: Supplement 1. — Trial Protocol [file jamanetwopen-e2332715-s001.pdf]

This supplement contains the following items:

- Study Protocol and Statistical Analysis Plan (original and only version)
- Summary of Changes

Health Care Hotspotting: A Randomized Controlled Trial

Analysis Plan

Date: March 7, 2014

Abstract

This randomized trial investigates the impact of the Camden Coalition of Healthcare Providers' Care Management Program: Link2Care. The program targets "super-utilizers" of the health care system - specifically adults with 2 or more hospitalizations in the last six months and 2 or more chronic conditions - with intensive care-management services in the one to three months following hospital discharge. A team of nurses, social workers, community health workers and health coaches, supported by real-time data of healthcare utilization, perform home visits, accompany patients to doctor visits, and help patients enroll in social-service programs. This approach aims to improve the self-sufficiency of patients in navigating the healthcare and social-service systems and has the potential to reduce healthcare costs and improve patient health. Through random assignment to the intervention, we plan to study the impact of the Link2Care program, particularly on subsequent hospital use.

PRINCIPAL INVESTIGATOR: Jeffrey Brenner[1], MD and Amy Finkelstein[2], PhD

CO-INVESTIGATOR(S):

Joseph Doyle[3], PhD

Sarah Taubman[4], ScD

Ruohua Annetta Zhou[5], PhD candidate

DEPARTMENT(S):

1. Department of Family Medicine, Cooper Health System
2. Department of Economics, Massachusetts Institute of Technology
3. Sloan School of Management, Massachusetts Institute of Technology
4. National Bureau of Economic Research
5. PhD Program in Health Policy, Harvard University

1. Introduction

This document details our analysis plan for: Health Care Hotspotting: A Randomized Controlled Trial. This will serve as a record of the planned analyses to avoid concerns over searching for results *ex post*. We anticipate that further analyses will be conducted as more outcome data become available, which we describe below, as well as research that is inspired by the initial findings. In addition, as the plan has been prepared in advance of the pilot, it is possible that our planned analyses may be modified based on what we learn during the pilot.

The structure of the plan is as follows: Section 2 briefly describes the intervention; Section 3 reviews the related literature and highlights the innovations of this intervention; Section 4 provides details on the trial itself; Section 5 describes the data and the primary outcome of interest; Section 6 presents the main empirical models, including Tables and Figures that we will complete as part of the study; Section 7 reports preliminary analyses in support of the plan; Section 8 briefly outlines our planned next steps in terms of additional data gathering and analyses; and Section 9 concludes with caveats and interpretation issues.

2. The intervention

The Camden Coalition of Healthcare Providers' Care Management Program, Link2Care, targets "super-utilizers" of the health care system. These are individuals with medically and socially complex needs who have frequent hospital admissions. Specifically, the Link2Care program targets patients in Camden, New Jersey hospitals who have had at least two hospital admissions in the prior six months and have at least two chronic conditions.

Such heavy utilizers of hospital care account for a disproportionate share of healthcare spending. For example, CCHP analyzed hospital admission and emergency department use at three Camden hospital systems from 2002-2007 and found that 20% of patients accounted for 90% of the costs (Green et al., 2010). When we compare patients admitted to Camden hospitals, in the year prior to an admission a typical patient targeted by the program has 2.5 times more admissions in the prior six months compared to other patients due to the targeting. Such patients are also much more likely to be readmitted to the hospital over the year following the hospital stay, accruing $73,000 in hospital charges over that time compared to an average of $6600 for patients not targeted by the program.

In an effort to improve the health of these patients and reduce subsequent healthcare costs, Link2Care provides intensive care management and coordination for up to 6 months following hospital discharge. Participants are assigned to a multidisciplinary care team comprised of a registered nurse, a licensed practical nurse, a social worker, an intervention specialist, a community health worker, and health coaches. They then plan a series of home visits, schedule and accompany patients to initial primary care and specialty care visits, and support individuals

as they navigate various social service agencies to enroll in public programs including Medicaid, SNAP/TANF, substance abuse and behavioral health programs, and programs that promote housing stability.

More specifically, upon discharge from the hospital, the care team works to visit the patient at home within 3 days. At the initial home visit, the care team (1) performs medication reconciliation—an inventory of the medications prescribed to gauge appropriateness and patient understanding, (2) conducts an assessment of the patient's perception of the discharge experience and care coordination, medical/health needs, activity/mobility, service needs, and stage of readiness to change, and (3) collaboratively sets goals with the individual, such as compliance with the discharge plan.

The care team then works closely with the patient to achieve these goals. They aid in scheduling a primary care visit within 7 days of discharge and appropriate specialist visits, as necessary. A member of the team will accompany the patient to those visits to ensure that such appointments are kept and that the patient understands any instructions. The team also helps the participant complete applications for social services and coaches the patient in self-care. Subsequent home visits evaluate the patient's and the team's progress. The end of the intervention is determined based on hospital utilization, individual factors (health education/literacy, disease self-management, skills development, level of engagement, self-efficacy) and some systemic factors (access to, and the quality of, care, social support, etc.). The person receives a graduation certificate and is then expected to meet healthcare needs in the future through a primary care physician. From October 2012 to January 2014, the median length of the intervention for those who completed it was 85 days.

The approach aims to improve the self-sufficiency of patients in navigating the healthcare and social-service systems. It has the potential to reduce healthcare costs and improve patient health, as patients learn to use primary care to prevent an escalation of symptoms that leads to rehospitalization. In an earlier, non-randomized evaluation, this program has been found to improve health outcomes, decrease utilization of emergency and inpatient services, and decrease costs for a cohort of 36 "high utilizers" from $1.2 million monthly to $534,000 monthly, a savings of 56% over five years (Green *et al.*, 2010).

Due to staffing and financial constraints, Link2Care is currently administered for only a subset of the patients who meet the eligibility criteria, and the patients who are currently approached are chosen in an ad-hoc manner. This study would establish a formal process for determining – via random assignment – which subset of eligible individuals are offered the intervention. This random assignment, which will not reduce the number of individuals who benefit from the services, will allow us to isolate the causal effects of the CCHP Link2Care Program.

These findings will not only be of importance to CCHP and the Camden patients they serve, but will also inform any expansion of CCHP's programs. The Link2Care program has received

national attention (Gawande 2011). And the CCHP's 2012 Center for Medicare and Medicaid Innovation grant has funded four new sites that are implementing the Link2Care model in Allentown, Pennsylvania, Aurora, Colorado, Kansas City, Missouri, and San Diego, California.

3. Related literature

3.1. Brief literature review

Transitional care programs come in a variety of forms, and there is compelling evidence that these interventions can have large effects on subsequent utilization. Naylor et al. (2011) reviewed the literature on these programs. They studied 21 RCTs and found 9 that showed significant reductions in readmissions. In our own review of nine RCTs of care management programs with at least two home visits by nurses, we also find that some programs are shown to significantly reduce readmissions but not all.[1] Takeda et al. (2012) reviewed 25 RCTs of disease management interventions for chronic heart failure (CHF) patients. They conclude that case management interventions led by a heart failure specialist nurse, as well as multidisciplinary interventions delivered by a team, are successful in reducing readmissions and mortality at twelve months.

While the evidence is therefore mixed, Naylor et al. point out that there were some common themes among the more-successful interventions. In particular, they tended to be engaged with patients for a longer period of time—typically 2-3 months—and they usually included home visits by nurses. In addition, they often included "comprehensive discharge planning with follow-up interventions that incorporate patient and caregiver goal setting, individualized care planning, educational and behavioral strategies, and clinical management." Link2Care aims to implement these best practices.

One of the more successful interventions is described by Naylor et al. (2004): a three-month intervention with nurse home visits for a population over the age of 65 with congestive heart failure. The treatment group had a rehospitalization or death at 52 weeks at a rate of 47.5% vs. 61.2% for the control group, a difference that was statistically significant with a sample size of 239.

In terms of heterogeneity of effects, prior studies often lack the power to distinguish effects across subgroups. Some studies (e.g. Shumway et al. 2008 and Jack et al. 2009) found a larger reduction in subsequent utilization among "high risk" subgroups, identified as either having higher utilization prior to the intervention or more co-morbidities. Studies that focus on mental-health patients tend to find little effect of interventions. Link2Care targets patients who may be

[1] Rich et al. (1995), Naylor et al. (1999), Naylor et al. (2004), Daly et al. (2005), and Parry (2009) find significant reductions in subsequent utilization; Siu et al., (1996), Brown et al. (2006), Allen et al. (2009), and Dixon et al. (2009) do not find such reductions.

the most amenable to such an intervention: high-cost patients who suffer from multiple comorbidities, excluding patients who suffer exclusively from mental health conditions.

3.2. Distinguishing features of Link2Care

A few main characteristics distinguish Link2Care from interventions in the existing literature. First, the Link2Care model specifically targets "super-utilizers" of hospital care. Patients have to have had 2 or more admissions in the past 6 months to qualify for the program. A few studies targeted high-utilizers of emergency departments (Shumway et al. 2008, Spillane et al. 1996), but this is a very different (and lower cost) population; we have not seen any other randomized evaluation of an intervention that targets hospital "high-utilizers" explicitly.[2] Given the right-skewness of health care spending, an intervention that can "bend the cost curve" for the highest cost patients would be of great interest.

Second, it is unique in the sense that it has both an intensive clinical component and an intensive social component. The typical transitional care intervention tends to be more focused on the clinical component, such as designing an individualized care plan, discussing patients' needs, assisting patients in making follow-up appointments and sometimes accompanying them to these appointments. Link2Care teams seem to make an exceptionally intense and aggressive effort to help patients obtain social services. For example, the intervention team not only helps patients apply for housing, but accompanies them to the Social Security Administration and the Motor Vehicle Commission offices to obtain necessary identification.

One intervention that appears to have similarly intensive social interventions as Link2Care was conducted by Shumway et al. (2008). This intervention did not include a clinical component, however. It did target patients who frequented the emergency department and found a significant reduction in emergency department visits between 19 and 24 months after discharge. Other studies mention social workers used as part of the case management, but do not focus on their role.

Third, whereas most existing studies focus on Medicare patients, VA patients, or patients with specific chronic disease diagnoses, there is no such restriction on the targeted population for Link2Care. The only requirement is that patients need to have some sort of insurance in order to be eligible. As a result, Link2Care is offered to a wide range of patients: patients with different diagnoses; adults of all ages, and Spanish-speaking individuals (who are typically excluded from similar studies).

4. Trial details

[2] It is worth noting that while none of the other studies specifically focus on high-utilizers, their targeted populations, which are typically enrolled at hospital bedside before discharge, may still have a high hospitalization rate in general.

4.1. Enrollment

Enrollment in the trial will be conducted by CCHP in a process similar to the one currently used to enroll patients into Link2Care. Patients will be enrolled at the two major Camden hospitals: Cooper University Hospital and Our Lady of Lourdes.

Identification of eligible patients will rely on a Health Information Exchange (HIE) that CCHP helped to develop. The hospital systems currently in the HIE include Cooper, Lourdes, and Virtua (a local hospital system with an emergency department in Camden). CCHP is currently exploring adding two more nearby hospitals, Kennedy Hospital and Underwood Memorial Hospital, to the HIE.

CCHP receives a daily data feed of individuals currently admitted to Cooper or Lourdes hospitals who have had two or more hospital admissions in the prior six months to any of the hospitals in the HIE. A staff member reviews the HIE along with electronic medical record data for pre-specified inclusion and exclusion criteria.

Patients must satisfy the following criteria based on the records at the time of the index event:

- Is currently admitted to Cooper or Lourdes hospitals (still in hospital for recruitment)
- Is 19-80 years old
- Resides in the following ZIP codes: 08101 (PO ZIP code), 08102-08105, 08107
- Has >=2 hospital admissions in the past 6 months (to hospitals in the HIE[3])
- Has >=2 chronic conditions

Patients must meet at least three of the following criteria based largely on the electronic medical record:

- Has >=5 outpatient medications
- Has difficulty accessing services
- Lacks social support
- Has a mental health co-morbidity
- Is actively using drugs
- Is homeless

Patients will be excluded if they meet any of the following criteria:

- Already a subject in the RCT (treatment or control)
- Deceased or discharged prior to triage or recruitment
- Uninsured
- Cognitively impaired
- Oncology patient
- Index hospitalization is for:

[3] This currently includes Cooper (1 hospital), Lourdes (2 hospitals), and Virtua (4 hospitals), but will change if additional hospitals are added to the HIE.

- A surgical procedure for an acute problem
- Complications of a progressive chronic disease with limited treatments
- A mental health issue only with no co-morbid conditions

A CCHP recruiter will approach individuals deemed eligible before the candidate is discharged from the hospital. The recruiter will explain the purpose, process, risks and benefits of the study, including the randomization process and intervention. An informed consent form will be provided and explained. Candidates can choose to enroll in the trial by signing the consent form. If the candidate enrolls, the recruiter will conduct a short survey for which the subjects will be compensated $20 for their time. Only after the completion of the survey will the recruiter learn the results of the randomization and inform the patient.

If an individual is randomized to the intervention, a care management team will be assigned to the case. For individuals randomized to control, they will receive the usual standard of care, but not the additional CCHP intervention. The standard of care in this case includes a printed hospital discharge plan prior to leaving the hospital.

4.2. Timing

The trial will be piloted beginning in March or April 2014, and we anticipate recruiting patients at least through June 2015. We plan to continue enrollment until we attain at least 800 subjects (400 treatment, 400 controls), as described in the next section.

5. Data sources and outcomes

5.1. Data sources

This plan details the initial analyses using administrative claims data recorded from emergency department and inpatient stays in Camden-area hospitals that are members of the Health Information Exchange (HIE) described above. These data can be matched to study participants using identifiers including the subject's name, SSN, date of birth, gender and address. The data include typical claims-data elements including detailed information about admission and discharge dates, diagnosis and procedure codes, insurance status and hospital charges and receipts.

The flow of how the HIE is used to identify subjects, monitor their enrollment, and study their outcomes is described by the following figure:

Data Flow

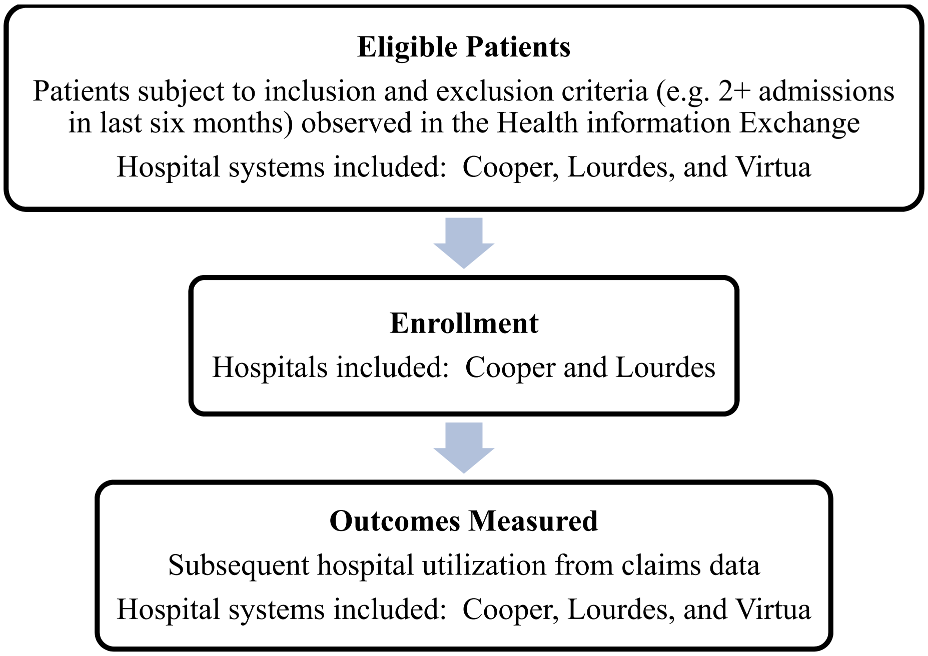

Below we describe analyses of the comprehensive nature of these data sources and our pursuit of complementary data sources.

5.2. Primary outcome

The primary outcome we will analyze is whether Link2Care reduces hospital readmissions, as patients become more self-sufficient in securing medical care outside of the hospital. In particular, our primary outcome is any readmission within 180 days of discharge from the hospital stay when the subject was enrolled in the study. We will follow the CMS convention of not counting a transfer from the hospital where the patient was enrolled to another hospital as a readmission, as such a transfer would apply to the current episode and the patient would not be at risk of entering the Link2Care Program, which begins once the patient returns home.

5.3. Secondary outcomes

Other outcomes of interest include measures of healthcare utilization, including emergency department (ED) visits, and treatment intensity associated with readmissions (number of days, number of procedures, hospital charges, and hospital receipts). We are interested in measuring effects in the short-term while the program is actively providing services (30-days, 90-days), and longer-term outcomes (180-days, 365 days).

5.4. Power calculations and expected sample size

We employed historical discharge data from the two enrolling hospitals from 2003-2010 to perform power calculations. First, we limited the sample to patients who are similar to those targeted by Link2Care: two or more admissions in the prior six months, between the ages of 19 and 80, two chronic conditions recorded in the diagnosis codes in the prior year, the chronic conditions could not all be mental-health conditions, and the patient was not diagnosed with cancer in the prior year. Note that this approximates the inclusion/exclusion criteria used when determining eligibility through the HIE described above, but it does not include the criteria found in the electronic medical record.

We determined that with 80% power and a test size of 0.05, a sample size of 800 would provide the statistical power to detect a 30% reduction in our primary outcome: 180-day hospital readmission. Compared to the mean 180-day readmission rate of 0.30, this means that we would have the power to detect a drop to 0.21. Although this is a large effect, it is within the set of estimates found in the literature for intensive case management featuring home visits described in the literature review.

Another 180-day outcome where we would have the power to detect a 30% decline is the likelihood of 2 or more "hospital visits", defined as outpatient ED visits plus inpatient readmissions, which averages 0.32. Other 180-day outcomes where we have the power to detect a 20% change include any hospital visit (an outcome that averages 0.54) and the likelihood of any outpatient ED visit (an average of 0.42).[4]

If we have the power to detect a given percentage change in an outcome at six months, we have the power to detect an effect at twelve months as well (e.g. 365-day readmission). Other outcomes where we have the power to detect a 30% decline over the following 365 days include the number of hospital visits (which averages 2.7) and the likelihood of 3 or more visits (which averages 0.33).

As a result of these power calculations, we plan to recruit until we attain a sample size of 800 subjects. Based on historical data on the number of eligible patients and the program capacity, we expect this will take 1-2 years. CCHP is considering options that may increase the sample size of the study or the speed at which the target is reached (such as expanding to nearby ZIP codes).

6. Empirical model and planned tables

[4] For the power calculations we could only observe outpatient ED visits, but we expect to observe all ED visits in the future. Given the higher means associated with all ED visits, we will have more power to detect a given percentage change in this measure.

6.1. Empirical model

The main analysis will compare outcomes for those who were randomized to receive Link2Care compared to those who were randomized to the control group.

Consider an outcome, Y, such as an indicator that the patient was readmitted to a hospital within 180 days after discharge from the hospital. For subject i, the estimating equation is:

$$Y_i = \beta_0 + \beta_1 1(\text{Treatment})_i + \beta_2 X_i + \varepsilon_i$$

where $1(\text{Treatment})_i$ is an indicator variable equal to one if the subject was randomized to the treatment group and zero if the subject was randomized to the control group; X_i is a vector of control variables. These control variables should be uncorrelated with the treatment indicator, but they can aid in the precision of the estimate. We propose standard demographic and prior-utilization controls: age (5-year age bins), sex (indicator that the patient is male), race/ethnicity (indicators for African American and Hispanic), and measures of utilization in the 0-6 months prior to the enrollment admission and 7-12 months prior to the enrollment admission corresponding to the outcome in the model.[5] For example, if the outcome is a readmission indicator, the prior-utilization controls will include the number of admissions over those time periods; if it is a hospital visit (inpatient readmission or emergency department visit), the prior-utilization controls will describe the number of such visits, and for hospital charges and receipts they will be the totals for those measures.

β_1 is the parameter of interest and measures the Intent to Treat: the causal effect of being randomized into the treatment group. This can differ from the effect of the program if there is non-compliance: some subjects in the treatment group may not be treated and/or subjects in the control group may be treated. For example, among those assigned to treatment, a patient who is transferred from the hospital to a long-term-care facility will no longer be eligible for home visits and Link2Care will never begin, or some subjects could withdraw from the study prior to treatment.[6] We expect take up to be high, however, because it is currently close to 100% for those approached to be part of Link2Care, and we are randomizing subjects into treatment after they have consented to be part of the study. We anticipate that subjects assigned to the control group will not participate in the program by design.

To the extent that there is non-compliance, we will also estimate the effect of actually participating in Link2Care, using the random assignment into the treatment group as an instrumental variable for program participation. That is, we will estimate for subject i:

---

[5] In our power calculations, we considered variables that reduced the residual variation in the readmission outcomes, and we discovered that the number of admissions in the 0-6 months prior to admission for the index event, the number of admissions in the 7-12 months prior to the admission for the index event, age, and sex were useful in this regard. We did not have the race/ethnicity variable available in our analysis dataset.

[6] We do not anticipate that the program will affect such transfers, but we can investigate by considering the rate at which subjects are discharged home.

$$Y_i = \pi_0 + \pi_1 1(\text{Enrolled in Link2Care})_i + \pi_2 X_i + \vartheta_i$$

This model will be estimated using Two-Stage Least Squares (2SLS), where the first stage is:

$$1(\text{Enrolled in Link2Care})_i = \alpha_0 + \alpha_1 1(\text{Treatment})_i + \alpha_2 X_i + \omega_i$$

The resulting estimate of π_1 is the local average treatment effect: the average treatment effect for those induced into program participation due to the random assignment into the treatment group.

We will also consider time to readmission in days, which can be right-censored if the person has not been readmitted by the time of the analysis. First, we will estimate Kaplan-Meier survival graphs associated with time to readmission for the treatment vs. the control group. We will also estimate a Cox proportional hazard model for subject i:

$$h(t)_i = h_0(t) \exp\{\beta_1 1(\text{Treatment})_i + \beta_2 X_i\}$$

where $\exp\{\beta_1\}$ is the hazard ratio of interest.

6.2. Planned tables and figures

The following tables list (1) variables that are available in the claims data from the hospitals that are part of the HIE, (2) data that we expect to capture in a baseline survey, and (3) data on the treatment intensity of the program that will be collected by CCHP. Some of these variables we have not used in our preliminary analysis, and although we expect these standard variables will be of high quality, we cannot attest to the quality of every variable at this time.

6.2.1. Means comparison using administrative claims data

Table 1 reports means of the characteristics of patients in the administrative claims data. Column 1 reports means for all inpatients admitted to the enrolling hospitals during our study period. For each individual admitted over the trial period, we will randomly select one of their admissions to compare to the remaining columns.

Column 2 reports means for all patients who meet the inclusion/exclusion criteria for the program. The comparison of Columns 1 and 2 will highlight the extent to which the program targets high-cost patients.

Our estimates provide a causal effect of treatment among the group of potential subjects who agree to be part of the study. A natural question is how representative this group is compared to other high-cost patients who are less willing to participate. Columns 3 and 4 compare those who are targeted for the intervention but do not agree to be part of the trial to those who do agree to participate. The refusal rate will be the number of observations in the refused column (3) divided the number of observations in the targeted column (2).

Columns 5 and 6 compare the patients who are randomized into the treatment and the control groups. This provides a check on whether the groups are similar to one another, as expected due to the randomization. Column 7 reports the p-value from a test that the means in Columns 5 and 6 are equal to one another.

The first set of rows includes demographic characteristics including age, sex, race and ethnicity. We also observe the ZIP code of residence, which will be used to characterize the median household income of the area where the patient lives.

We then characterize the index admission—the admission where the subjects are enrolled—including the hospital of admission, and whether the admission type was categorized as an emergency. Intensity of treatment at the index admission, such as length of stay, could, in principle, be affected by the treatment. This will be described in Appendix Table 1 rather than in this table, which is meant to consider the balance of observables across the treatment and control groups.

From the longitudinal discharge data, we can also observe hospital utilization in the year prior to the trial, and we will compare various summary measures across the groups. We will also compare the primary payer, defined as the payer responsible for most hospital payments over the prior year.

6.2.2. Means comparisons of trial subjects at baseline

In addition to the discharge data, upon consenting to be in the trial the enrollment staff conducts an intake survey. Table 2 describes the variables that are available in the baseline survey and proposes to collapse some of the answers into categories for the sake of brevity. In particular, the survey asks questions about the usual place where patients receive medical care, marital status and family support (which may be important here as Link2Care enlists the family to help the patient gain self-sufficiency), military service history (to aid in understanding what programs the patient might be eligible to receive), living arrangements, literacy, educational attainment and employment. Two self-reported health questions are asked, as well as questions that verify the presence of certain conditions, including mental-health conditions, obesity, and substance abuse.

To summarize the information collected at the time the participants are targeted, and from the baseline survey, CCHP calculates illness-severity scores, and we can compare these scores across the groups as well.

6.2.3. Outcomes

Table 3 presents the results for the primary outcome: an indicator that the patient was readmitted to a hospital in the claims data within 180 days from discharge from the enrolling hospital.

To complement the analysis of the primary outcome, Figure 1 presents another way to characterize the time to a readmission: the Kaplan-Meier estimate of the survival function. For each day, D, this will plot the fraction of each group that has "survived" at least D days without a readmission. At zero days both groups will have a probability of one, and the survival functions will fall as patients are readmitted over time.

Of course we are interested in other outcomes as well, and although the power to detect differences for some of these outcomes is lower than the primary outcome, we may find effects that can yield insights into the sources of the main effects and inspire future research.

Table 4 presents the results for secondary outcomes and other outcomes of interest. Panel A of Table 4 includes estimates of effects on other types of hospital use within the same 180 days of discharge: any use of an emergency department and the combined outcome of any emergency department visit or inpatient readmission; as well as inpatient readmissions that are from the emergency department and those that are not from the emergency department, as it is possible that the program prevents emergency readmissions but may promote scheduled readmissions. We will also measure the intensity of hospital utilization by considering the number of readmissions, the likelihood of two or more readmissions, the number of days in the hospital, hospital charges, and hospital receipts. Hospital charges and receipts reflect treatment intensity, but also reflect pricing decisions by hospitals and efforts to receive payment, which will impact the interpretation of the findings. They are typically skewed as well, and we will report effects of the program on the median of these measures in robustness checks described below.

We are interested in whether effects are found during the intervention versus longer-term outcomes where the power to detect effects is somewhat higher. Panel B shows that we plan to consider 30-, 90- and 365-day measures of hospital use, as well as days to readmission estimated with a Cox proportional hazard model.

Different types of patients may experience different casual effects of the program. After consulting the literature and practitioners, Panel C describes subsets that we would like to investigate separately. These include patients with more admissions in the prior year, as the literature suggests that effects may be larger for higher-cost patients: we can consider whether this relationship continues to be found within this set of high-cost patients. In our examination of historical claims data, 70.4% of patients likely targeted by the program had 3 or more visits in the prior year. One innovation in this study is that we consider a large non-English speaking population in addition to English-speaking patients, and we can report differences in treatment effects across these two groups.

Other subgroups of interest from the literature and the nature of the intervention include those who reported substance abuse, psychiatric disorders, and low family support at the baseline survey. We will consider such groups if the subgroups represent a substantial share of patients, a calculation we will perform once we have the baseline survey results. Although we are likely

underpowered to detect differences in effects across groups, we consider such comparisons important precursors for future research.

6.2.4. Program participation and treatment intensity

Tables 3 and 4 report intent-to-treat effects associated with assignment to the treatment group. Table 5 describes the extent to which the treatment and control groups received CCHP services.

First, program intensity is explored by reporting the means across the treatment and control groups in terms of: whether the subject was assigned to treatment—expected to be 1 for treatment and 0 for controls as a check on any contamination. Next, whether Link2Care was initiated will be considered. Then we will report the length of the intervention in days and the number of home visits, which will include zeroes for subjects where Link2Care was not initiated. These estimates should be close to zero for the control group by design.

The next set of characteristics provides some information on why Link2Care may not have been initiated. Patients who enter a long-term-care facility are not suitable for Link2Care, and this is not always known at the time of enrollment. The claims dataset includes a discharge status, which reports whether the patient was discharged home or was transferred to another facility, which means the subject may begin the program but only once they return home. The discharge status also records whether the patient expired in the hospital.

For the treatment group, we will know more about adherence to the goals of Link2Care, which we will report in Panel C: whether a visit to a PCP was achieved within 7 days, whether the patient visited a specialist if that was part of the discharge plan, aid in enrolling in welfare programs, whether transportation for appointments was arranged, enrollment in a medical day program for substance abuse or behavioral health, obtained an official identification or power of attorney, and whether the team advocated for the client over bills, court warrants, etc. We will also report which of the two Link2Care teams the subject was assigned.

6.2.5. Robustness checks

We will report empirical estimates using alternative empirical specifications, largely as a check on the robustness of the main results.

First, Appendix Table 2 provides robustness checks for the primary-outcome result shown in Table 3, including estimates with and without controls, and estimates from a logit model. In addition, estimates will be presented from instrumental variable (2SLS) estimation, which allows us to estimate the effect of Link2Care for those induced into the program due to the assignment to the treatment group: a local average treatment effect (LATE). This will take into account the fact that some of those in the treatment group never enroll in Link2Care and cases where patients in the control group were somehow treated.

Appendix Tables 3A and 3B repeat the comparisons made in Table 4, but check for robustness to the estimator used (e.g. the logit model), the exclusion of controls, and the LATE estimates. Days to readmission is not considered in these tables, as this measure is censored.

Appendix Table 4 reports intent-to-treat and LATE estimates for three hospital utilization outcomes: inpatient readmission, any emergency department visit, and any hospital use (readmission or ED visit). The estimates will be shown across four time horizons: 30, 90, 180, and 365 days.

7. Preliminary analyses

7.1. Who are super-utilizers? Historical data comparisons

We analyzed a limited dataset of claims data from 2002-2011 available to CCHP for the two main Camden hospitals: Cooper and Lourdes. These data contain a subset of the variables we will access in the future. Consistent with the inclusion criteria, we limited the data to patients who are between ages 19 and 80 at the index admission and who have not been diagnosed with cancer.

We then identified "super utilizers"—our proxy for patients likely targeted by Link2Care—as individuals who at any point had two admissions within a 6 month period (the second of these was designated as the index admission), and had at least 2 chronic conditions that were not exclusively mental health related.

For patients with more than one admission, we randomly selected an index admission between 2003 and 2009. This ensures one year of pre- and 2 years of post-admission data.

As can be seen from Appendix Table 5, the targeted patients tend to be older, less likely to be female, tend to come from ZIP codes with lower household incomes. By design, they also had higher hospital use in the year prior to the index admissions: 2.8 inpatient admissions accruing close to $200,000 in hospital charges and $25,000 in hospital receipts. These levels are 2.5 to 4 times higher than other patients admitted to these hospitals, with 1.1 admissions, $50,000 in charges and $6,000 in receipts. These differences did not converge in the year following the admission. Rather, typical patients targeted by the program have 0.9 readmissions and 2.6 visits to the hospital when we include the emergency department. Other patients have only 0.09 readmissions on average, and 1 visit to the hospital. In terms of hospital charges and receipts, those targeted by the program receive care that totals $73,000 in charges ($8500 in receipts) compared to $6600 in charges ($773 in receipts) for other patients.

7.2. Camden-area hospital coverage

The longitudinal discharge data that we will access includes information for the three main hospital systems in the Camden area: Cooper, Lourdes, and Virtua. It is of course possible for subjects to be admitted to other hospitals. To the extent that the intervention impacts hospital choice, this could lead to different rates of Camden-area hospital readmissions between the treatment and control groups that could be unrepresentative of effects on readmissions to any hospital.

To investigate this issue, we studied discharge data from New Jersey in 2011. Although these data are not longitudinal in nature, they do allow us to identify the ZIP code of residence of patients admitted to each hospital. When we consider admissions of patients who reside in the ZIP codes where CCHP recruits patients, we find that 87.2% are admitted to Cooper, Lourdes, and Virtua hospitals. Outside of these three hospital systems, the hospitals with the greatest share of patients from these ZIP codes are Kennedy Hospital, with 10.6%, and Underwood Hospital, with 0.43%. As noted above, we are hopeful that the longitudinal discharge data will include these hospitals in the near future, which would bring the coverage rate up to 98.2%.[7] The next largest share is attributed to Capital Hospital, at 0.41%, which is located in Trenton, NJ: 36 miles north of Cooper Hospital.

Of course, it is also possible that patients are admitted to hospitals in other states, including hospitals across the Delaware River in Pennsylvania. We are current working to obtain discharge data from that state to see how often Camden-area residents are admitted to those hospitals. Further, we hope to consider other longitudinal data that includes all hospitals, as described in the next section.

8. Planned next steps

This plan focuses on analyses using longitudinal discharge data from the main hospitals in Camden, NJ. We are currently working to gain access to other data sources that will provide additional outcomes of interest.

8.1. Centers for Medicare and Medicaid Services (CMS) data

To complement the claims data from the three hospital systems, other measures of utilization may become available from the Centers for Medicare and Medicaid Services (CMS). In particular, we are interested in enrollment into Medicaid, as this is often facilitated by Link2Care. While Link2Care targets individuals who are insured, some are eligible for Medicaid but have not yet enrolled.

---

[7] For patients recruited from ZIP codes that are almost entirely within the Camden city limits (08102, 08103, 08104, 08105), Cooper, Lourdes and Virtua hospitals admit 92.6% of patients, and adding Kennedy and Underwood increases the coverage to 98.3%.

For subjects who are covered by Medicare and/or Medicaid at the start of the study, we hope to study healthcare utilization. First, we can revisit the readmission variable using all hospitals rather than the ones in our initial set. Second, we can analyze outpatient utilization, which provides a broader picture of the costs of the program as well as potential mechanisms by which the program may reduce re-hospitalization.

In particular, a goal of the program is to ensure that each patient sees a primary care physician with seven days after discharge and specialists soon after discharge if that is part of the discharge plan. We hope to measure the rate at which this happens, as well as the number of physician visits over time.

We anticipate that one mechanism for reducing re-hospitalization is closer monitoring of prescription medication, and we hope to measure the number of prescriptions filled and changes to the prescription regimen over time.

Admission to a skilled nursing facility is costly, and we hope to use CMS data to estimate the effects of program participation on this outcome as well.

In terms of a health outcome, we plan to consider is mortality, which is available from CMS data. We believe mortality rates are not as high among this population that spans the entire adult age range compared to prior work that focused on the elderly, however.

8.2 Social services data

An innovation of the program is the coaching of subjects to navigate the social-services system to enroll in programs that they are eligible to receive. We are investigating a variety of data sources to measure these outcomes. From the NJ Department of Human Services, we hope to link the subjects to program participation data for TANF and SNAP programs. From the NJ Department of Labor and the Pennsylvania Department of Labor, we hope to link subjects to unemployment insurance records to measure employment and wages. From the NJ Department of Corrections, we hope to measure incarceration rates. Homelessness services and housing stability programs administered by the Camden Housing Authority would allow us to measure participation as a potential mechanism for reducing hospital utilization. We are also investigating other sources of mortality data, including Social Security Administration data and NJ Vital Statistics data.

9. Interpretation

This trial holds the potential to evaluate the value of the Link2Care program in terms of subsequent hospital utilization, and provide insights into the types of patients who are most affected by Link2Care.

One caveat is that the program may have other benefits and costs that we are not currently measuring. For example, Link2Care aims to improve health and quality of life. We will attempt to measure these with mortality, healthcare utilization associated with greater limitations using the CMS data, and social-services data such as employment measures.

Link2Care may also have costs beyond operational costs, including the greater use of outpatient care and prescription drugs. Again, we aim to expand the data sources to include longitudinal data on all healthcare use.

Second, the study will consider a program that is well established. If we find large effects, it will be useful to investigate how the lessons learned by CCHP can be replicated in other areas.

Third, the study considers the entire package of services provided by the Link2Care program. It will not be possible to ascertain which components of the program are most effective or whether the impact is greater because they are offered together. Subsequent research could consider the components separately.

Fourth, the study is particularly appropriate to study "partial equilibrium" effects of Link2Care. If Link2Care were to greatly expand and dramatically affect hospital revenues, this could have broader effects that this study is not designed to estimate.

References

Allen K, Hazelett S, Jarjoura, D, Hua Keding, Wright K, Weinhardt J, Kropp D. A Randomized Trial Testing the Superiority of a Postdischarge Care Management Model for Stroke Survivors. *J Stroke Cerebrovasc Dis*. 2009 18(6):443-452.

Brown MD, Reeves MJ, Meyerson K, Korzeniewski SJ. Randomized Trial of a Comprehensive Asthma Education Program after an Emergency Department Visti. *Ann Allergy Asthma Immunol.* 2006; 97:44-51.

Daly BJ, Douglas SL, Genet Kelley C, O'Toole E, Montenegro H. Trial of a Disease Management Program to Reduce Hospital Readmissions of the Chronically Ill. *Chest*. 2005 128(2):507-517.

Dixon L, Goldberg R, Iannone V, Lucksted A, Brown C, Kreyenbuhl J, Fang L, Potts W. Use of a Critical Time Intervention to Promote Continuity of Care After Psychiatric Inpatient Hospitalization. *Psychiatric Services* 2009 60(4): 451-458.

Gawande, Atul. "The Hot Spotters: Can we lower medical costs by giving the neediest patients better care?" *The New Yorker*. January 24, 2011. http://www.newyorker.com/reporting/2011/01/24/110124fa_fact_gawande

Green, Steven; Singh, V; O'Byrne, W. Hope for New Jer's City Hospitals: The Camden Initiative . *Perspectives in Health Information Management*, Spring 2010.

Jack BW, Chetty VK, Anthony D, et al. A reengineered hospital discharge program to decrease rehospitalization: a randomized trial. Ann Intern Med 2009;150:178-87.

Naylor MD, Brooten D, Campbell R, Jacobsen BS, Mezey MD, Pauly MV, Schwartz JS. Comprehensive Discharge Planning and Home Follow-Up of Hospitalized Elders: A Randomized Clinical Trial. JAMA 1999; 281(7): 613-620.

Naylor MD, Brooten D, Campbell R, *et al*., "Transitional care of older adults hospitalized with heart failure: a randomized, controlled trial." *J Am Geriatr Soc*, 2004 May;52(5):675-84.

Naylor MD, Aiken LH, Kurtzman ET, Olds DM, Hirschman KB. The care span: The importance of transitional care in achieving health reform. Health Aff (Millwood) 2011;30:746-54.

Parry C, Min SJ, Chugh A, Chalmers S, Coleman EA. Further Application of the Care Transitions Intervention: Results of a Randomized Controlled Trial Conducted in a Fee-For-Service Setting. *Home Health Care Services Quarterly*. 2009. 28(2-3):84-99.

Rich, MW, Beckham V, Wittenberg C, Leven CL, Freedland KE, Carney RM. A Multidisciplinary Intervention to Prevent the Readmission of Elderly Patients with Congestive Heart Failure. *New England Journal of Medicine*, 1995 333(18): 1190-1195.

Shumway M, Boccellari A, O'Brien K, Okin RL. Cost-effectiveness of clinical case management for ED frequent users: results of a randomized trial. Am J Emerg Med 2008;26:155-64.

Siu AL, Kravitz RL, Keeler E, Hemmerling K, Kington R, Davis JW, Mitchell A, Burton TM, Morgenstern H, Beers MH, Reuben DB. Postdischarge Geriatric Assessment of Hospitalized Frail Elderly Patients. *Arch Intern Med* 1996; 156:76-81.

Spillane LL, Lumb EW, Cobaugh DJ, Wilcox SR, Clark JS, Schneider SM. Frequent users of the emergency department: can we intervene? Acad Emerg Med 1997;4:574-80.

Takeda A, Taylor SJ, Taylor RS, Khan F, Krum H, Underwood M. Clinical service organisation for heart failure. Cochrane Database Syst Rev 2012;9:CD002752.

Table 1: Means Comparisons

	All Inpatients	Targeted	Targeted: Refused	Targeted: Consented	Consented: Treatment	Consented: Control	p-value
	(1)	(2)	(3)	(4)	(5)	(6)	(7)
Demographics							
Age							
Age under 35							
Age 35-44							
Age 45-54							
Age 55-64							
Age 65-74							
Age 75+							
Male							
White							
African American							
Hispanic							
ZIP Code: Median HH Income							
Index-admission characteristics:							
Admitted to Cooper Hospital							
Admission type: Emergency							
Healthcare utilization in year prior to the admission:							
Number of admissions							
Number of emergency department visits							
Number of days in the hospital							
Number of procedures							
Hospital charges							
Hospital payments							
Primary payer							
Medicaid							
Medicare							
Dual-eligible for Medicaid and Medicare							
Private insurance							
Self-pay							
Observations							

Source: Claims data from hospitals in the Health Information Exchange. Column (1) includes all patients admitted to Cooper and Lourdes Hospitals--the recruitment sources for the trial. The primary payer will be determined by the payer responsible for the largest dollar-amount of claims over the year. Column 7 reports the p-value for the null hypothesis that the means in columns (5) and (6) are equal to one another.

Table 2: Baseline Survey Comparison: Treatment vs. Control

		Treatment (1)	Control (2)	p-value (3)
Existing primary care	Has a Primary Care Physician (PCP)			
	Conditional on having a PCP: Months since last visit			
Most common place for care:	Doctor's office			
	Emergency room/Urgent care clinic			
	Other			
Marital status	Married/Civil Union/Cohabitating			
	Single/Divorced/Widowed			
	Other/prefer not to say			
Social network: Family/friend supports healthcare needs	Never/rarely			
	Sometimes			
	Mostly/Always			
	Unable to respond			
Military service	Active or Veteran			
Living arrangements	Own house/apartment/room			
	With a friend/relative			
	Shelter/boarding home/streets			
	Other/prefer not to say			
Language/Literacy	Preferred language: English			
	Preferred language: Spanish			
	Preferred method to learn new information includes Reading			
Educational Attainment	Grades 6-8			
	Grades 9-12			
	GED			
	HS diploma			
	Some College/Associates Degree			
	Bachelor's degree +			
	Other/prefer not to say			
Employment	Currently employed			
	Refused/Don't know			
Self-reported health status during past 12 months	Excellent			
	Very Good			
	Good			
	Fair			
	Poor			
	Must stay in bed/need help of another person getting around			
	Not limited in any way			
Common conditions	Mental health condition			
	Congestive Heart Failure			
	COPD/Emphysema			
	Coronary Artery Disease			
	Diabetes			
	HIV/AIDS			
	Obesity			
	Substance Abuse			
	Other recorded condition			
CCHP aggregation	Pre-enrollment severity score from targeting info			
	Pre-enrollment severity score from baseline survey			
	Observations			

Source: Baseline Survey. Column 3 reports the p-value for the null hypothesis that the means in columns (1) and (2) are equal to one another.

Table 3: Main Outcome: 180-day Readmission

Dependent Variable:	Control group mean (1)	Intent to Treat (2)
180-day any hospital readmission		
Observations		

Source: Claims data from hospitals in the Health Information Exchange. The intent to treat effect estimate comes from an OLS regression that includes controls for controls for age (5-year age bins), sex (indicator that the patient is male), race/ethnicity (indicators for African American and Hispanic), the number of admissions in the 0-6 months and 7-12 months prior to the enrollment admission, as described in the text. Standard errors in parentheses. * significant at 10%; ** significant at 5%; *** significant at 1%.

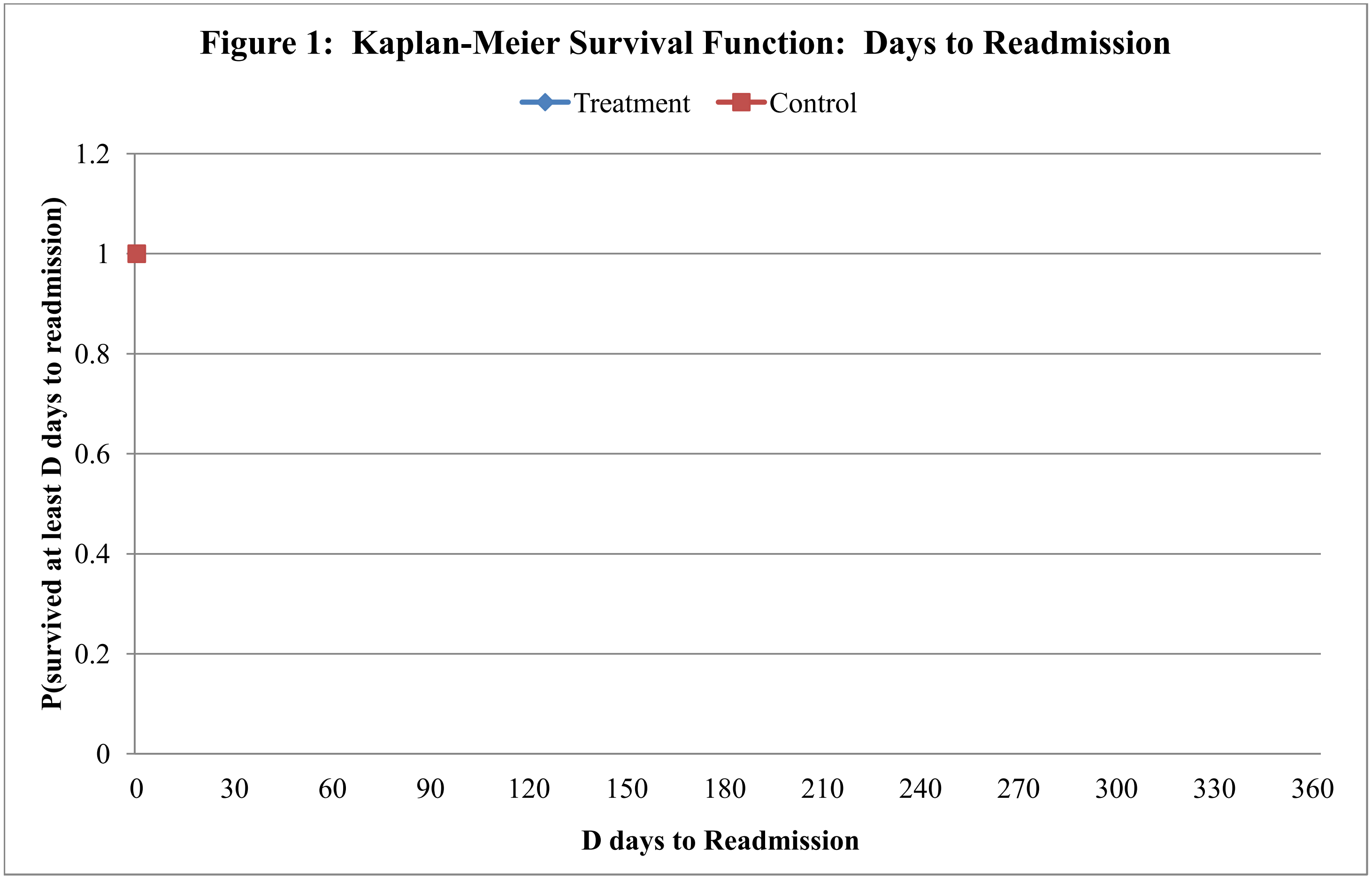
Figure 1: Kaplan-Meier Survival Function: Days to Readmission
Treatment
Control
P(survived at least D days to readmission)
1.2
1
0.8
0.6
0.4
0.2
0
0
30
60
90
120
150
180
210
240
270
300
330
360
D days to Readmission

Table 4: Further Outcomes and Subgroups

		Control group mean (1)	Intent to Treat (2)
**A. Additional 180-day Outcomes**			
	Dependent Variable:		
Hospital visits	Any emergency department (ED) use		
	Any hospital use (inpatient or ED)		
	Inpatient readmission from the ED		
	Inpatient readmission not from the ED		
Treatment intensity	Number of readmissions		
	2+ readmissions		
	Number of days in the hospital		
	Hospital charges		
	Hospital receipts		
**B. Time horizons**			
	30-day readmission		
	90-day readmission		
	365-day readmission		
	365-day any hospital use (inpatient or ED)		
	Time to readmission (days): hazard ratio		
**C. Subgroups**			
	Dependent Variable: 180-day readmission		
	Patients with 3+ readmissions in the prior year		
	Patients with 2 readmissions in the prior year		
	English speaking		
	Non-English speaking		
	Observations		

Source: Claims data from hospitals in the Health Information Exchange. Time to readmission hazard ratio is estimated with Cox proportional hazard model; all other models are estimated using Ordinary Least Squares. All models include controls for age (5-year age bins), sex (indicator that the patient is male), race/ethnicity (indicators for African American and Hispanic), the corresponding 0-6 month and 7-12 month prior-utilization controls as described in the text. Standard errors in parentheses.
* significant at 10%; ** significant at 5%; *** significant at 1% for a test that the coefficient equals zero or the hazard ratio equals one.

Table 5: Program Participation & Intensity

	Treatment (1)	Control (2)	p-value (3)
**A. Program intensity**			
Assigned to treatment group			
Enrolled in Link2Care			
Length of the intervention (days)			
Number of home visits			
**B. Discharge status from index admission**			
home			
transferred to another facility			
expired			

C. Link2Care Goals and Services Provided

Dependent Variable:	Treatment (1)
Visited PCP within 7 days of returning home	
Visited specialist within 30 days as part of discharge plan	
Enrolled in Medicaid	
Enrolled in SNAP/TANF	
Arranged transportation	
Secured official identification/power of attorney	
Enrolled in medical day programs (substance abuse & behavioral health)	
Enrolled in disability/SSI	
Enrolled in housing stability program	
Advocated for patient (bills/court warrants/etc.)	
Assigned to Team A	
Observations	

Source: Panels A and C: CCHP program data; Panel B: Claims data from hospitals in the Health Information Exchange. Column 3 reports the p-value for the null hypothesis that the means in columns (1) and (2) are equal to one another.

Appendix Table 1: Hospital Utilization at Index Admission

	Treatment (1)	Control (2)	p-value (3)
Length of stay in hospital			
Number of procedures			
Hospital charges			
Hospital payments			
Observations			

Source: Claims data from hospitals in the Health Information Exchange. Column 3 reports the p-value for the null hypothesis that the means in columns (1) and (2) are equal to one another.

Appendix Table 2: Checks for Main Outcome: 180-day Readmission

Dependent Variable: 180-day any hospital readmission

A. Intent to Treat

Model	Intent to Treat (1)	(2)
OLS		
Logit		

B. Local average treatment effect (LATE)

Model	LATE (1)	(2)
2SLS		
Controls	No	Yes
Control group mean		
Observations		

Source: Claims data from hospitals in the Health Information Exchange. Panel A includes estimates from a logit model, and the average marginal effect is reported. Controls are those desribed in Table 3. Panel C is estimated using 2SLS, and the parameter estimate represents the average treatment effect for those iduced to participate in the program as a result of the random assignment to the treatment group. Standard errors in parentheses. * significant at 10%; ** significant at 5%; *** significant at 1%.

Appendix Table 3A: Further Outcome Checks: Alternative Estimation Models

		Model	Control group mean (1)	Intent to Treat Estimate (2)
**A. Additional 180-day Outcomes**				
	Dependent Variable:			
Hospital visits	Any emergency department (ED) use	Logit		
	Any hospital use (inpatient or ED)	Logit		
	Inpatient readmission from the ED	Logit		
	Inpatient readmission not from the ED	Logit		
Treatment intensity	Number of readmissions	Poisson		
	2+ readmissions	Logit		
	Number of days in the hospital	Poisson		
	Hospital charges	Median regression		
	Hospital receipts	Median regression		
**B. Time horizons**				
	30-day readmission	Logit		
	90-day readmission	Logit		
	365-day readmission	Logit		
	365-day any hospital use (inpatient or ED)	Logit		
**C. Subgroups**				
	Dependent Variable: 180-day readmission			
	Patients with 3+ readmissions in the prior year	Logit		
	Patients with 2 readmissions in the prior year	Logit		
	English speaking	Logit		
	Non-English speaking	Logit		
	Observations			

Source: Claims data from hospitals in the Health Information Exchange. This table includes the analyses with controls as in Table 4 but using alternative estimators. Average marginal effects are reported. Standard errors in parentheses. * significant at 10%; ** significant at 5%; *** significant at 1%.

Appendix Table 3B: Further Outcomes and Sub-groups: LATE

		Control group mean	Intent to Treat		LATE	
		(1)	(2)	(3)	(4)	(5)
**A. Additional 180-day Outcomes**						
	Dependent Variable:					
Hospital visits	Any emergency department (ED) use					
	Any hospital use (inpatient or ED)					
	Inpatient readmission from the ED					
	Inpatient readmission not from the ED					
Treatment intensity	Number of readmissions					
	2+ readmissions					
	Number of days in the hospital					
	Hospital charges					
	Hospital receipts					
**B. Time horizons**						
	30-day readmission					
	90-day readmission					
	365-day readmission					
	365-day any hospital use (inpatient or ED)					
**C. Subgroups**						
	Dependent Variable: 180-day readmission					
	Patients with 3+ readmissions in the prior year					
	Patients with 2 readmissions in the prior year					
	English speaking					
	Non-English speaking					
	Controls		No	Yes	No	Yes
	Observations					

Source: Claims data from hospitals in the Health Information Exchange. This table includes OLS and 2SLS estimates with controls correspoding to the analyses in Table 4. For the 2SLS results, the parameter estimates represent the local average treatment effect (LATE) for those iduced to participate in the program as a result of the random assignment to the treatment group. Standard errors in parentheses. * significant at 10%; ** significant at 5%; *** significant at 1%.

Appendix Table 4: Time Horizons for Additional Outcomes

Dependent Variable:	Intent to Treat (1)	Intent to Treat (2)	LATE (3)	LATE (4)
Inpatient Readmission				
30-day				
90-day				
180-day				
365-day				
Any emergency department (ED) use				
30-day				
90-day				
180-day				
365-day				
Any hospital use (inpatient or ED)				
30-day				
90-day				
180-day				
365-day				
Controls	No	Yes	No	Yes
Observations				

Source: Claims data from hospitals in the Health Information Exchange. This table includes OLS estimates with controls similar to Table 4. Standard errors in parentheses. * significant at 10%; ** significant at 5%; *** significant at 1%.

Appendix Table 5: All Inpatients vs. Targeted Inpatients

		All Inpatients (1)	Not Targeted (2)	Targeted (3)
Demographics	Age	42.88	40.31	53.55
	Female	0.62	0.65	0.53
Median Income	ZIP Code: Distribution of top 5 most common			
$23,719	08102	0.10	0.10	0.12
$27,980	08103	0.21	0.20	0.22
$24,631	08104	0.26	0.26	0.25
$31,896	08105	0.30	0.31	0.27
$51,599	08110	0.08	0.08	0.08
Hospital use in the year prior to the index admission				
	Number of admissions	1.43	1.1	2.81
	Number of ED visits, not-admitted	1.25	1.05	2.04
	Number of hospital visits (admitted or ED)	2.68	2.15	4.85
	Hospital charges	79212	50277	198896
	Hospital receipts	9644	5975	24822
Hospital use after the index admission				
Number of hospital readmissions	within 6 months	0.14	0.05	0.52
	within 12 months	0.24	0.09	0.87
	within 24 months	0.43	0.19	1.4
Number of hospital visits (admitted or ED)	within 6 months	0.71	0.54	1.47
	within 12 months	1.31	1.01	2.55
	within 24 months	2.38	1.9	4.33
Hospital charges	within 6 months	11150	3205	44011
	within 12 months	19594	6603	73332
	within 24 months	32946	13139	114875
Hospital receipts	within 6 months	1303	375	5139
	within 12 months	2269	773	8460
	within 24 months	3863	1592	13257
	Observations	25574	20595	4979

Source: Historical claims data from Cooper Hospital and Our Lady of Lourdes Hospital admitted between 2003 and 2009. Sample is restricted to patients between the ages of 19 and 80 who have not been diagnosed with cancer. Each observation is a randomly selected admission for an individual admitted to these hospitals. Our proxy for "targeted" using claims data are those with two admissions within a 6 month period and had at least 2 chronic conditions that were not exclusively mental-health related.

Summary of Changes

The trial protocol and planned analyses were designed (and publicly pre-specified) in March 2014. The trial protocol was faithfully implemented.

Recruitment ran longer than expected in order to reach the target of 800 participants. We had planned to run recruitment from March or April 2014 through at least June 2015. In fact, we piloted from March 29, 2014 to May 30, 2014, and the study population was enrolled from June 2, 2014 through September 13, 2017.

The planned analyses were followed quite closely: in particular the specification (including the pre-specified controls), the primary outcome, the pre-specified subgroup analysis of the primary outcome and most of the secondary analyses were all implemented as pre-specified. We document here the specific departures from our analysis plan.

Pre-specified analyses that were not executed.

1. We were unable to obtain reliable measures of Emergency Department (ED) use with the currently available data. Therefore, we have not analyzed secondary outcomes that involve ED use (specifically: any ED visit, any ED or hospital visit, any inpatient readmission from the ED, and any inpatient readmissions not from the ED).
2. We estimate the sensitivity of the analysis of the primary outcome to excluding covariates and to use Logit instead of OLS analysis (see Table S6) as pre-specified. However, we did not re-estimate any of the secondary outcomes excluding covariates or using a Logit as pre-specified, as it made no difference for the primary outcome.
3. We pre-specified as robustness analyses a Poisson analysis of several secondary outcomes (number of readmissions, number of days in the hospital) and a median regression of several secondary outcomes (charges and receipts). Given the null results on all the main primary and secondary outcomes, we chose not to do additional analyses.
4. We pre-specified a 365-day Kaplan-Meier survival curve analysis but instead implement a 180-day Kaplan-Meier survival curve analysis (Figure S4) because data were not yet available for 365 days for the whole sample.
5. In addition to the primary intent-to-treat specification presented here, we pre-specified additional analyses in which we would estimate two-stage least squares models in which we used assignment to treatment (vs. control) as an instrument for assignment received. This was pre-specified in case some of those in the treatment group ended up not enrolled in the program or some in the control group ended up treated. In practice, however, none of the control group were treated and all of the intervention attempted to reach all of the treatment group. Since an exact definition of what it means to succeed in that attempt (i.e. enrollment) is not clear, we did not in the end implement any two-stage least squares analyses. Instead, we simply present the program engagement metrics in Table 2.

6. For the analysis of program engagement metrics, the set of metrics examined in Table 2 differs somewhat from what was pre-specified. At the time of pre-specification the data were not available, and we subsequently learned that several measures were not available or alternative measures were available that made more sense.
7. The balance tests in Tables 1 and S2 differ slightly from what we pre-specified. In particular, Table 1 shows balance on all the pre-specified controls, but we did not pre-specify that we would test balance on all of them. In addition, in Table S2 for outcomes with more than two discrete possibilities, we reported the share in the category with the most mass (while we pre-specified that we would report them all, which created many more rows). We also report index admission diagnoses from the discharge data rather than self-reported common conditions from the baseline survey (we use the discharge data whenever possible in the analysis, as it is more granular and perhaps more accurate than the self-reports in the baseline survey). For completeness, in Tables S9 and S10 we show the pre-specified balance tests, excluding only variables we ended up being unable to measure (namely variables related to ED use and procedures).
8. There are two cases where the questions asked in the Baseline Survey administered in practice differ slightly from the version that was pre-specified.
    a. In the Demographics section of the Baseline Survey, we pre-specified a different set of answer choices for race and ethnicity where Hispanic/Latino as an answer choice for ethnicity but not a choice for race. In practice, the question actually asked to participants regarding race allowed for Hispanic/Latino to be an answer. This is reflected in Table 1.
    b. In the Health Status & Support section of the Baseline Survey, we pre-specified a question about family supporting healthcare needs. We pre-specified one of the answer choices be "often," in practice, this answer choice was "mostly." This change is reflected in Table S10.

Analyses that were not pre-specified.

We conducted a few additional analyses that were not pre-specified:

1. Table 3: We added unadjusted comparisons of means for treatment and control groups.
2. Mean reversion analyses (Figures 2 and S3 and Table S5) were not pre-specified.
3. Analysis of social services receipts (SNAP, TANF and GA) in Table 2; at the time we wrote the pre-analysis plan we were not sure whether we would obtain those data.
4. Table S6: We added analysis measuring the 180 days from the time of admission rather than discharge.
5. Mortality analysis and competing risks analysis (Table S8)
6. The additional heterogeneity analysis in Figure S5
7. The sensitivity to excluding data from Kennedy Hospital in Table S6.
8. Comparison of study population to general population (Table S4).
