# Supplementary material for: Hospital Readmissions by Variation in Engagement in the Health Care Hotspotting Trial: A Secondary Analysis of a Randomized Clinical Trial
Source: JAMA Netw Open. 2023 Sep 12;6(9):e2332715. doi: 10.1001/jamanetworkopen.2023.32715 (PMC10498327; doi:10.1001/jamanetworkopen.2023.32715)
Supplement: Supplement 2. — eAppendix 1. Data sources eAppendix 2. Sensitivity analysis of engaged participation definition eAppendix 3. Model building eAppendix 4. Poisson model validations eAppendix 5. Additional analysis eTable. Intervention and control group patient characteristics within increasingly distilled samples eFigure. Number of intervention hours received by treatment arm patients during their first week of intervention enrollment eReferences [file jamanetwopen-e2332715-s002.pdf]

Supplemental Online Content

Yang Q, Wiest D, Davis AC, Truchil A, Adams JL. Hospital readmissions by variation in engagement in the Health Care Hotspotting trial: a secondary analysis of a randomized clinical trial. *JAMA Netw Open*. 2023;6(9):e2332715. doi:10.1001/jamanetworkopen.2023.32715

eAppendix 1. Data sources

eAppendix 2. Sensitivity analysis of engaged participation definition

eAppendix 3. Model building

eAppendix 4. Poisson model validations

eAppendix 5. Additional analysis

eTable 1. Intervention and control group patient characteristics within increasingly distilled samples

eFigure 1. Number of intervention hours received by treatment arm patients during their first week of intervention enrollment

eReferences

This supplemental material has been provided by the authors to give readers additional information about their work.

eAppendix 1. Data sources

Camden Health Information Exchange:

The Camden Health Information Exchange (HIE), developed and operated by the Camden Coalition, is a real-time, web-based portal and database that unifies patient data from a variety of clinical providers across southern New Jersey, including the region's three major health systems: Cooper, Virtua, and Jefferson Health. The HIE is operated on CareEvolution's™ platform and includes admission, discharge, transfer data for emergency department (ED) and inpatient encounters. The Camden Coalition used the HIE's real-time reporting function to identify patients who were eligible for the program and recruit them at their hospital bedside. The HIE data is a real-time database and not primarily a research database and is prone to concerns about the cleanliness and completeness of the data. While the research team modeled readmission outcome variables using the HIE data, additional data sources, including claims data and statewide uniform billing data, were secured to validate the information obtained through the HIE, described in A2.

Hospital discharge data (all-payer hospital claims)

The primary analytic data are hospital claims data through March 31, 2018 from the hospital systems described above. The data are at the hospital encounter level and contain demographic data, payer information, encounter types (e.g., inpatient or ED), admission and discharge dates, as well as diagnosis codes (ICD9 and ICD10). We matched these data to patients' admission records from the Camden HIE and labeled those hospital records as the index admission record for each patient. The index admission record was considered the baseline record; patients' clinical characteristics were captured from the diagnosis codes associated with the record.

The hospital claims data that were used in the new analysis had been revised and updated since the primary analysis was published.[1] Chi-square tests were used to test the independence between each outcome and the hospital claims data used in the two analyses. We found no significant difference across the treatment and control groups for each of the 30-, 90-, and 180-day readmission measures.

Comparison of readmission outcomes measured in primary and new analyses

	Primary analysis		New analysis		
	**Treatment group**	**Control group**	**Treatment group**	**Control group**	**P-value[a]**
180-day rate	62.3%	61.7%	60.1%	61.7%	0.82
90-day rate	49.1%	50.4%	47.1%	50.6%	0.80
30-day rate	28.2%	30.6%	26.7%	30.1%	0.91
180-day count (mean)	1.52	1.54	1.45	1.48	0.96
90-day count (mean)	0.94	0.92	0.88	0.89	0.82
30-day count (mean)	0.38	0.39	0.35	0.38	0.83

a. Chi-square tests were performed to test the independence between each outcome and the hospital claims data used in the two analyses.

© 2023 Yang Q et al. *JAMA Network Open.*

Care Coordination database

Camden Coalition used its internal workflow database, built in TrackVia™, to record programmatic information including the enrollment and randomization steps, and all post-enrollment workflows, intervention encounters, and staff effort. Two data tables were extracted for this study: baseline survey data and client tracking data. Camden Coalition care team staff completed the baseline survey during the initial visit with the patient at the hospital bedside before randomization or enrollment occurred. The survey covered demographic, clinical, social, and health-related self-assessment information. A client tracking table, where all staff-patient interactions including the interaction type, date, and hours spent, was used to calculate intervention-level measurements of participation and engagement.

Arrest history

The Camden Coalition entered into a data sharing agreement with the Camden County Police Department in 2014. The data shared by the police department with the Camden Coalition included name, date of birth, arrest date, location of arrest, and statute violation information for each arrest for the years 2014 through 2018. The arrest data were linked to the data described above.

© 2023 Yang Q et al. *JAMA Network Open.*

eAppendix 2. Sensitivity analysis of engaged participation definition

Intervention participation is a key concept in understanding program implementation and is a central component of the distillation method.[2] Because the Camden Core Model had no a priori criteria for "engaged participation," we developed a definition for the purpose of this analysis, as described in the main paper. In summary, we considered three dimensions of intervention activity to develop a definition:

1. Number of intervention hours received during the first two weeks of intervention enrollment
2. Number of weeks with a successful intervention encounter during the first six weeks of enrollment
3. Length of time between enrollment and intervention outcome (i.e., graduated or lost-to-follow-up)

The definition of engaged participation that we adopted is defined as anyone meeting at least two of the following criteria:

1. Received at least three intervention hours during the first two weeks of their enrollment
2. Had contact with staff at least once per week for four weeks out of the initial six weeks
3. Were retained in the program for 60 days (1/2 of average treatment length) or graduated within that timeframe

To better understand how robust the study findings are to other thresholds for defining engaged participation, we performed sensitivity analysis by varying the above three thresholds. The algorithm used for sensitivity analysis was as follows:

1. Let the number of intervention hours during the first two weeks run from its 20th percentile to 40th percentile, namely, it takes values from {1.8, 2.3, 2.7, 3.3}
2. For each value in a, let the number of weeks with a successful intervention encounter during the first six weeks run from its 20th percentile to 40th percentile, namely, taking values from {3, 4}
3. For each chosen values from a and b, let the number of days to intervention outcome runs from its 20th to 40th percentile, namely, taking values from {53, 58, 66, 85}

The 20th and 40th percentiles were chosen because many intervention patients actively participated in the program and graduated, but some patients received few intervention hours and were lost-to-follow-up soon after enrollment. These considerations suggested that the thresholds should not be set to the extremes of the distribution.

Percentiles for each component of engaged participation

	Engagement hours during intervention first two weeks	No. weeks out of first six with successful engagement	No. days to intervention outcome
0th	0.0	0.0	3.0
10th	0.8	1.2	34.3
20th	1.8	3.0	52.6
25th	2.3	3.0	58.3
30th	2.7	4.0	65.9
40th	3.3	4.0	85.2
50th	3.9	4.0	102.5
60th	4.7	5.0	125.0
70th	5.5	5.0	156.1
75th	6.0	5.0	163.0
80th	6.5	6.0	190.4
90th	8.5	6.0	259.8
100th	22.4	6.0	575.0

© 2023 Yang Q et al. *JAMA Network Open.*

The figures below plot the confidence intervals of odds ratios and incidence rate ratios for the 30-day and 180-day readmission rates and counts with varying parameter values of the engaged participation definition components. In each figure, the top 4 panels show the confidence intervals with a fixed number (3) of weeks with successful intervention encounters and a fixed number of days (53) to intervention outcome, but with varying intervention hours (1.8, 2.3, 2.7, 3.3) during the first two weeks. The number 5 and 6 panels in the middle show the confidence intervals with fixed number (1.8) of intervention hours and fixed number (53) of days to intervention outcome, but with a varying number of weeks (3, 4) with successful intervention encounters. Similarly, for the bottom 4 panels, the number of intervention hours and number of weeks with successful intervention encounters are fixed, the number of days to intervention outcome is varied. If the confidence interval is completely below or above the dotted horizontal line (y=1), then it is with solid circles to highlight significance.

For both the logistic and Poisson model analyses, the results are consistent with those reported in the main paper. For all models, downward trends are observed with increased population distillation and the treatment effect becomes statistically significant when engaged participation concentrations of the population are in the range of 40% to 20% of total participants. These sensitivity analyses can be interpreted to show that our results are robust to changes in the definition of engaged participation.

© 2023 Yang Q et al. *JAMA Network Open.*

Confidence intervals for odds ratio of the 30-day readmission rate at different levels of population distillation for varying values of each component of the engaged participation definition.

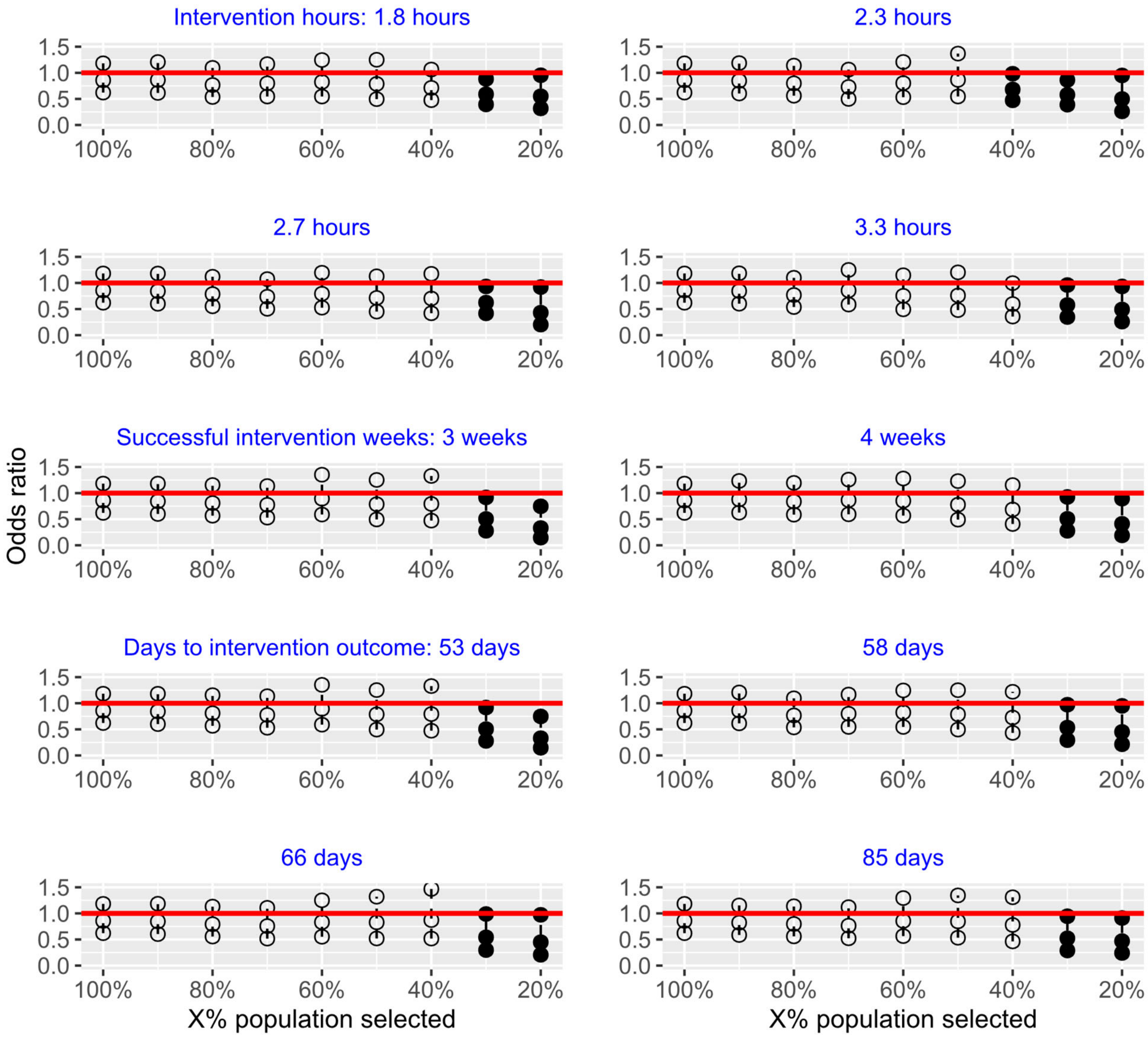

© 2023 Yang Q et al. *JAMA Network Open.*

Confidence intervals for odds ratio of the 180-day readmission rate at different levels of population distillation for varying values of each component of the engaged participation definition.

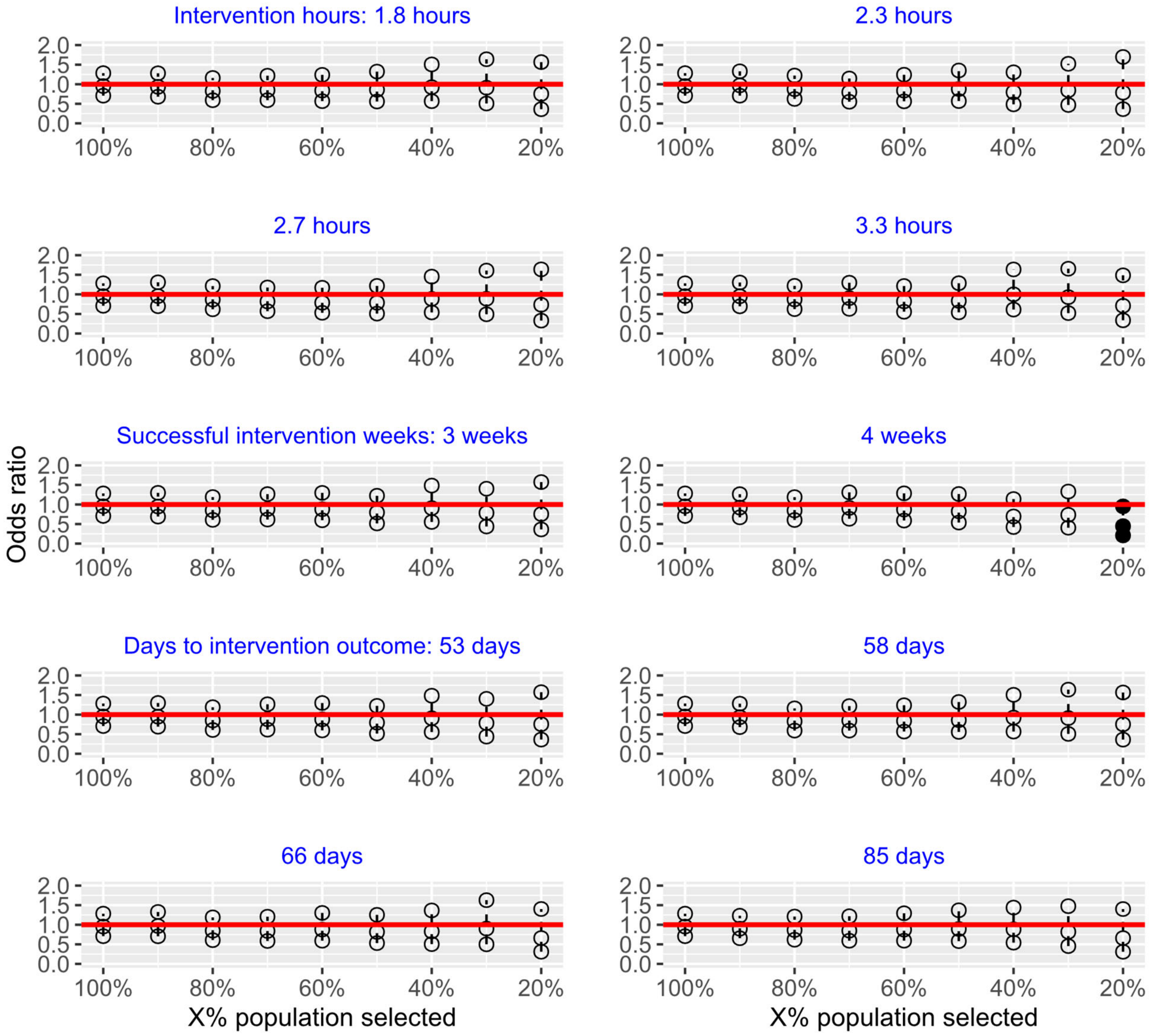

© 2023 Yang Q et al. *JAMA Network Open.*

Confidence intervals for incidence rate ratio of 30-day readmission counts at different levels of population distillation for varying values of each component of the engaged participation definition

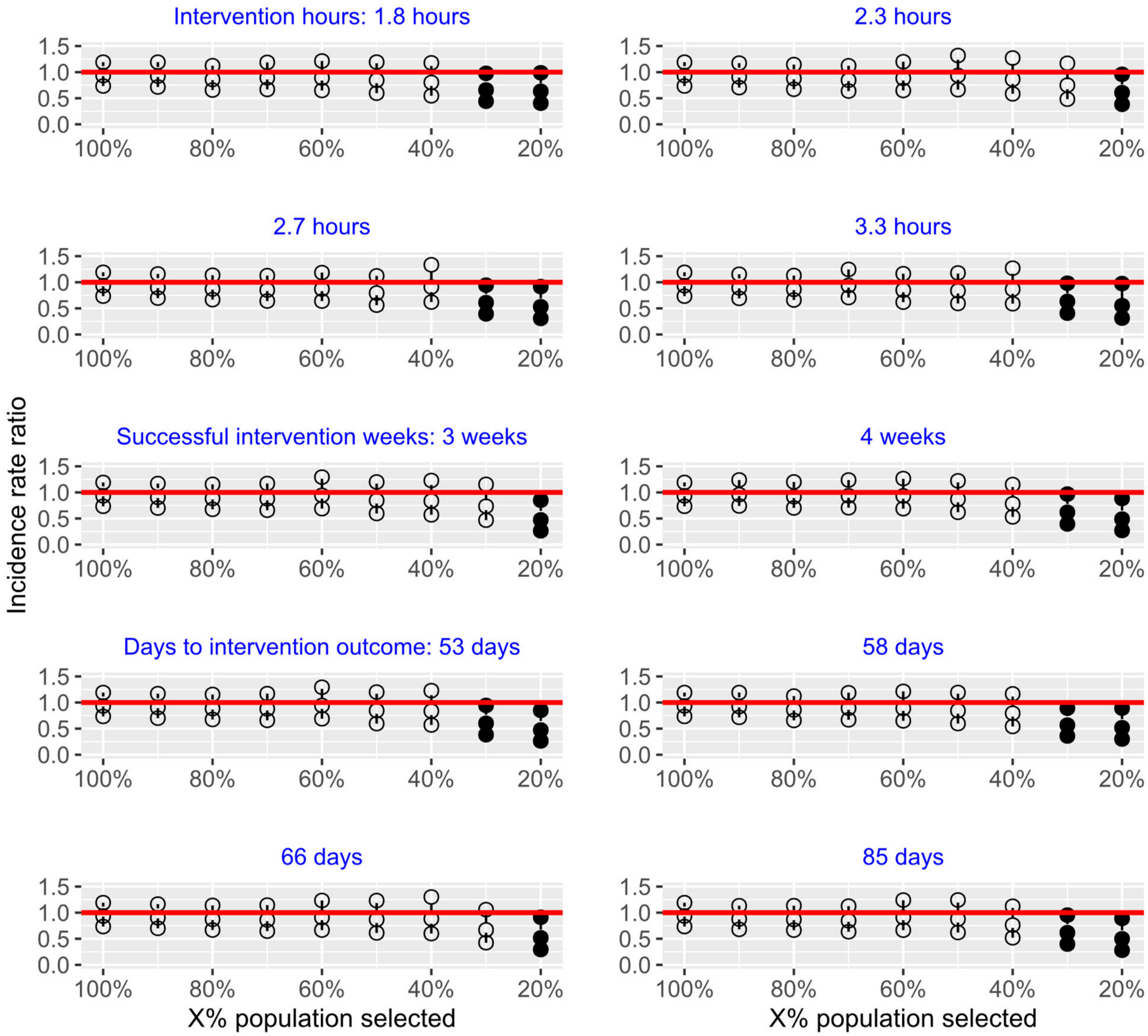

© 2023 Yang Q et al. *JAMA Network Open.*

Confidence intervals for incidence rate ratio of 180-day readmission counts at different levels of population distillation for varying values of each component of the engaged participation definition

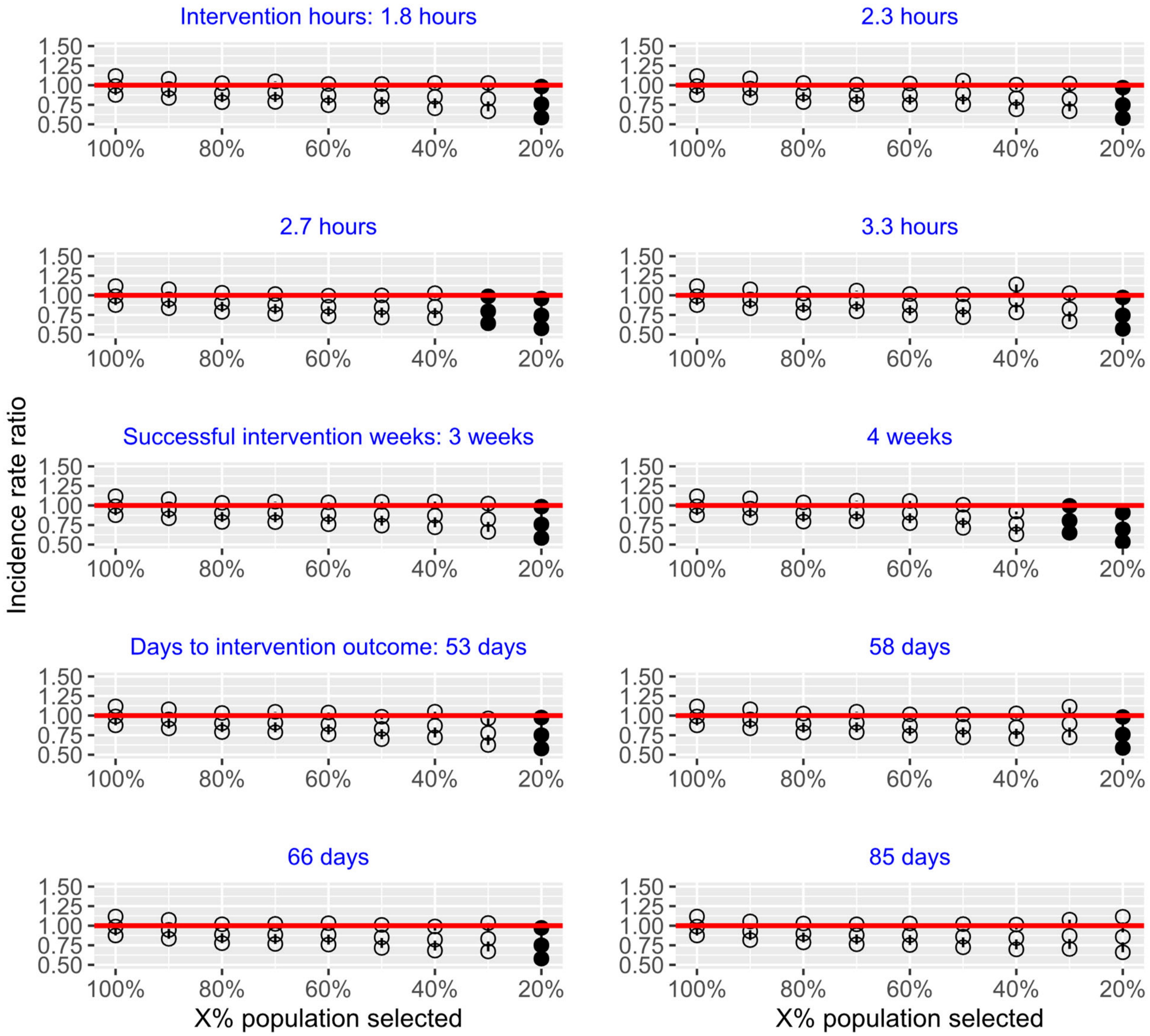

© 2023 Yang Q et al. *JAMA Network Open.*

eAppendix 3. Model building

Stage 1 Gradient boosting machine learning model

We built a gradient boosting machine model to estimate engaged participation among intervention patients. The variables included in the model are shown in the table below; all are drawn from the baseline period prior to enrollment.

Variables included in the gradient boosting machine model

Variable	Type	Range	Data source
Age	Numeric	18 - 80 years	Baseline survey
Gender	Categorical	Female, male	Baseline survey
Race & Ethnicity[a]	Categorical	Hispanic, Non-Hispanic Black, Non-Hispanic White	Baseline survey
Education level	Categorical	High school or higher degree; less than high school	Baseline survey
Marital status	Categorical	Married; not married, divorced, separated	Baseline survey
Housing status	Categorical	Stable; unstable	Baseline survey
Family support	Categorical	Sufficient; insufficient	Baseline survey
Employment	Categorical	Yes; no	Baseline survey
Any arrest in prior six months[b]	Categorical	Yes; no	Camden County Police Department
Self-reported heath status	Categorical	Poor; fair/good/excellent	Baseline survey
No. admissions in prior six months[c]	Numeric	2 - 18 admissions	Hospital claims
Index admission length of stay	Numeric	1 - 61 days	Hospital claims
Acquired immunodeficiency syndrome[d]	Categorical	Yes; no	Hospital claims
Chronic obstructive pulmonary disease	Categorical	Yes; no	Hospital claims
Chronic heart failure	Categorical	Yes; no	Hospital claims
Dementia	Categorical	Yes; no	Hospital claims
Diabetes with complication	Categorical	Yes; no	Hospital claims
Hemiplegia or paraplegia	Categorical	Yes; no	Hospital claims
Mild liver disease	Categorical	Yes; no	Hospital claims
Moderate or severe liver disease	Categorical	Yes; no	Hospital claims
Renal disease	Categorical	Yes; no	Hospital claims
Rheumatoid arthritis	Categorical	Yes; no	Hospital claims
Alcohol use diagnosis	Categorical	Yes; no	Hospital claims
Mental health diagnosis	Categorical	Yes; no	Hospital claims
Substance use diagnosis	Categorical	Yes; no	Hospital claims
Anxiety disorder	Categorical	Yes; no	Hospital claims
Mood disorder	Categorical	Yes; no	Hospital claims
Schizophrenia	Categorical	Yes; no	Hospital claims
Suicide ideation	Categorical	Yes; no	Hospital claims

a. Four patients indicated another race on the baseline survey (Asian, Multiracial, or Other). Because of modeling requirements, categories with only four elements could not be included in analysis. We therefore assigned these patients the most probable race and ethnicity category (Hispanic, Non-Hispanic Black, or Non-Hispanic White) such that the assigned category of each patient had the largest probability of association with their engaged participation outcome label.
b. Flags whether the patient had an arrest by the Camden County Police Department in the 6 months prior to trial enrollment.
c. Number of hospitalizations in 6 months prior to trial enrollment, including the index admission during which the patient was enrolled in the trial.
d. The medical diagnosis variables are diseases with a nonzero weight on the Quan index, which is an updated version of the Charlson co-morbidity index.[3]

© 2023 Yang Q et al. *JAMA Network Open.*

To find the optimal parameter values in the model, we used a line search algorithm defined as follows:

1. Learning rate runs from 0.001 to 0.01 with step increment 0.01
2. Number of observations allowed in one leaf runs from 5 to 10
3. Cross validation runs from 4-fold to 10-fold

The performance parameter is the area under the receiver operating characteristics curve (AUC-ROC). The optimal combination of parameter values are the values for which the AUC is maximized in the test data obtained from the cross validations. Cross validation was used to prevent overfitting. The optimal parameter values are learning rate 0.001, 8 observations allowed in each leaf, 8-fold cross validations, which achieve an AUC of 0.82. The variables added to the model to estimate engaged participation included demographic variables, clinical variables (physical illness, behavioral diagnosis indicators, and past hospitalizations), and social variables. Two-way interaction terms among variables were also added in the model.

Relative influence of variables predicting engaged participation based on gradient boosting machine model results

Variables	Relative influence value
Age	28.23
Index admission length of stay in days	9.00
No. admissions in prior six months	7.73
Housing status	7.50
Any arrest in prior six months	5.45
Race/ethnicity	4.95
Renal disease	4.21
Chronic obstructive pulmonary disease	3.64
Employment	3.25
Chronic heart failure	3.11
Education level	3.05
Mild liver disease	2.71
Self-reported heath status	2.24
Diabetes with complication	1.94
Mood disorder	2.02
Anxiety disorder	1.81
Gender	1.75
Family support	1.68
Suicide ideation	1.41
Alcohol disorder	1.38
Marital status	0.99
Substance use	1.34
Moderate or severe liver disease	0.63
Schizophrenia	0.33
Rheumatoid arthritis	0.11
Acquired immunodeficiency syndrome	0.05
Hemiplegia or paraplegia	0.00
Dementia	0.00

a. Higher relative influence values suggest a greater likelihood of being selected for classification by the model. b. The medical diagnosis variables are diseases with a nonzero weight on the Quan index.

© 2023 Yang Q et al. *JAMA Network Open.*

Stage Three Models Estimating Program Effects

We used logistic regression models to study 30-day, 90-day and 180-day readmission rates and Poisson regression models to study readmission counts. The formats of the independent variables and the inclusion of the variables in the final model were first determined based on bivariate analyses, then with a forward variable selection algorithm to select variables based on model performance metrics including the drop in AIC and/or p-values from Wald and likelihood ratio tests.[4,5]

Variables included in the regression models			
RCT group	Categorical	Intervention; control	Randomization process
Age 65+	Categorical	Yes; no	Baseline survey
Gender	Categorical	Female; male	Baseline survey
Race & Ethnicity	Categorical	Hispanic; non-Hispanic Black; non-Hispanic White	Baseline survey
Social scale[a]	Numeric	1-5	Baseline survey
Dichotomized Quan Index score[b]	Categorical	<3; 3+	Hospital claims
Mental health diagnosis indicator	Categorical	Yes; no	Hospital claims
Substance use diagnosis indictor	Categorical	Yes; no	Hospital claims
3+ admissions in prior six months	Categorical	Yes; no	Hospital claims

a. Each disease on the Quan Index has a value ranging from 0 to 6, where 0 means no mortality risk and 6 corresponds to the highest mortality risk. The variable used in our models aggregates scores across the diseases on the Quan Index. We then dichotomized this variable into two groups: score lower than 3 and score greater than or equal to 3.
b. The social scale is an aggregated score that measures a patients' social complexity. To create the scale, each of the following 5 attributes that were measured on the baseline survey was given a score of 1: less than a high school education, insufficient family support, unstably housed, unemployed, and unmarried (i.e., single, divorced, or widowed).

© 2023 Yang Q et al. *JAMA Network Open.*

eAppendix 4. Poisson regression model validation

The Poisson regression model has a strong assumption of equidisperson that needs to be validated before model application. We calculated the average 30-, 90-, and 180-day readmission counts and then divided these by their corresponding variance with the raw data, plotted as a pink dotted line in the figure below. To correctly apply Poisson models, we limited the dispersion of our data by winsorization. If a patient's readmission count was beyond the 95th percentile of the data, we cut the value at the 95th percentile. The variance-to-mean ratio after winsorization is plotted as a blue dotted line in figure below and was improved with winsorization.

Poisson regression model validations: variance-to-mean ratio

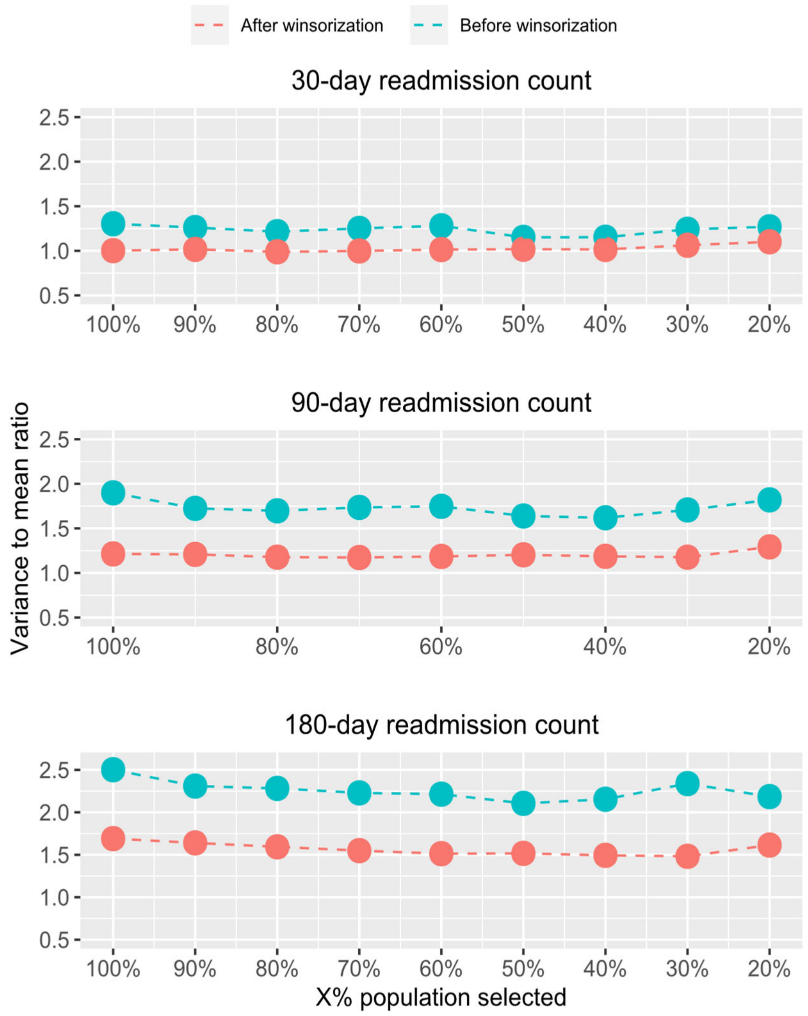

© 2023 Yang Q et al. *JAMA Network Open.*

To assess the integrity of the results after data winsorization, we reanalyzed the data using a robust sandwich estimator. The results from the two methods are similar and are shown in the table below.

Readmission results with Poisson regression models using winsorization and a robust sandwich estimator			
**Readmission outcome**	**X% of population selected**	**Adjusted incidence rate ratio (95% CI)**	
		Poisson regression with winsorization at 95th percentile	Poisson regression with robust sandwich estimator
30-day count	100	0.94 (0.73-1.19)	0.95 (0.75-1.20)
	60	0.93 (0.68-1.27)	0.96 (0.70-1.33)
	20	0.59 (0.36-0.96)	0.56 (0.32-0.98)
90-day count	100	0.99 (0.84-1.16)	1.00 (0.82-1.21)
	60	0.96 (0.78-1.18)	0.96 (0.75-1.22)
	20	0.80 (0.54-1.18)	0.80 (0.51-1.27)
180-day count	100	0.99 (0.88-1.12)	0.99 (0.83-1.18)
	60	0.88 (0.75-1.03)	0.85 (0.69-1.04)
	20	0.74 (0.56-0.99)	0.72 (0.52-0.997)

© 2023 Yang Q et al. *JAMA Network Open.*

eAppendix 5: Additional analysis

The primary results reported in the main paper are based on logistic and Poisson regression models. We also built Cox proportional hazard models to examine the time to first readmission. The data were right censored for patients with no hospital readmission within six months of their index hospital discharge. The first half of the table below displays the hazard ratio, the 95% confidence interval, and p-value based on the Cox models. The downward trend in the hazard ratio of intervention over control group becomes statistically significant at 20% population distillation (Hazard ratio: 0.64, 95% C.I. (0.42, 0.98), P=0.04).

Because Cox proportional hazard models only capture time to the first event, we built frailty models to capture the timing of repeated events. The second half of the table displays the hazard ratios, the 95% confidence intervals, and P-values at different levels of population distillation based on the frailty models. The declining hazard ratio becomes statistically significant at 20% distillation (Hazard ratio: 0.68, 95% C.I. (0.48, 0.97), P=0.03).

Both approaches yield results that are consistent with the main analyses presented in the main paper.

Hazard ratios of intervention over control group at different distillation levels			
X% of population selected	Hazard ratio	95% confidence interval	P-value
**Cox proportional hazard models**			
100	0.91	(0.76-1.09)	0.31
80	0.88	(0.72-1.08)	0.21
60	0.91	(0.72-1.14)	0.42
40	0.84	(0.64-1.12)	0.24
20	0.64	(0.42-0.98)	0.04
**Frailty repeated count models**			
100	1.05	(0.89-1.24)	0.55
80	1.10	(0.91-1.33)	0.32
60	0.94	(0.71-1.23)	0.63
40	0.85	(0.63-1.15)	0.30
20	0.68	(0.48-0.97)	0.03

© 2023 Yang Q et al. *JAMA Network Open.*

eTable 1: Intervention and control group patient characteristics within increasingly distilled samples

	Percentage of population selected									
	**100% (n=782)**		**80% (n=625)**		**60% (n=469)**		**40% (n=312)**		**20% (n=176)**	
	Treatment (n=393)	Control (n=389)	Treatment (n=311)	Control (n=314)	Treatment (n=244)	Control (n=225)	Treatment (n=162)	Control (n=150)	Treatment (n=79)	Control (n=77)
	**No. (%)**									
Age in years, mean [sd]	57.1 [12.5]	56.1 [12.8]	58.6 [10.9]	57.9 [11.1]	59.3 [9.6]	60.5 [8.9]	60.2 [8.8]	61.4 [6.9]	61.9 [7.4]	62.0 [6.0]
No. admissions in prior six months, mean [sd][a]	2.6 [1.6]	2.7 [1.6]	2.5 [1.3]	2.5 [1.2]	2.3 [1.0]	2.4 [1.1]	2.2 [0.6]	2.2 [1.0]	2.1 [0.3]	2.0 [0.5]
Index admission length of stay in days, mean [sd]	6.9 [5.4]	7.2 [6.2]	6.5 [4.5]	6.8 [5.1]	6.3 [3.9]	6.9 [4.6]	6.3 [3.9]	6.8 [4.6]	6.2 [3.5]	6.3 [3.2]
Male	204 (51.9)	183 (47.0)	155 (49.8)	138 (43.9)	121 (49.6)	96 (42.7)	77 (47.5)	58 (38.7)	35 (44.3)	26 (33.8)
Female	189 (48.1)	206 (53.0)	156 (50.2)	176 (56.1)	123 (50.4)	129 (57.3)	85 (52.5)	92 (61.3)	44 (55.7)	51 (66.2)
Hispanic	105 (26.7)	126 (32.4)	95 (30.5)	110 (35.0)	79 (32.4)	91 (40.4)	62 (38.3)	69 (46.0)	38 (48.1)	44 (57.1)
Non-Hispanic Black	226 (57.5)	201 (51.7)	177 (56.9)	165 (52.5)	138 (56.6)	116 (51.6)	89 (54.9)	73 (48.7)	38 (48.1)	32 (41.6)
Non-Hispanic White	62 (15.8)	62 (15.9)	39 (12.5)	39 (12.4)	27 (11.1)	18 (8.0)	11 (6.8)	8 (5.3)	3 (3.8)	1 (1.3)
At least a high school degree	209 (53.2)	210 (54.0)	155 (49.8)	162 (51.6)	116 (47.5)	99 (44.0)	64 (39.5)	50 (33.3)	21 (26.6)	15 (19.5)
Less than high school degree	184 (46.8)	179 (46.0)	156 (50.2)	152 (48.4)	128 (52.5)	126 (56.0)	98 (60.5)	100 (66.7)	58 (73.4)	62 (80.5)
Married/partnered	103 (26.2)	83 (21.3)	87 (28.0)	68 (21.7)	68 (27.9)	53 (23.6)	39 (24.1)	33 (22.0)	21 (26.6)	18 (23.4)
Single/divorced/widowed	290 (73.8)	306 (78.7)	225 (72.0)	246 (78.3)	176 (72.1)	172 (76.4)	123 (75.9)	117 (78.0)	58 (73.4)	59 (76.6)
Stably housed	355 (90.3)	350 (90.0)	294 (94.5)	291 (92.7)	230 (94.3)	212 (94.2)	154 (95.1)	142 (94.7)	77 (97.5)	73 (94.8)
Experiencing homelessness	38 (9.7)	39 (10.0)	17 (5.5)	23 (7.3)	14 (5.7)	13 (5.8)	8 (4.9)	8 (5.3)	2 (2.5)	4 (5.2)

© 2023 Yang Q et al. *JAMA Network Open.*

eTable 1: Intervention and control group patient characteristics within increasingly distilled samples										
	**Percentage of population selected**									
	**100% (n=782)**		**80% (n=625)**		**60% (n=469)**		**40% (n=312)**		**20% (n=176)**	
	Treatment (n=393)	Control (n=389)	Treatment (n=311)	Control (n=314)	Treatment (n=244)	Control (n=225)	Treatment (n=162)	Control (n=150)	Treatment (n=79)	Control (n=77)
	**No. (%)**									
Sufficient family support	244 (62.1)	224 (57.6)	198 (63.7)	195 (62.1)	153 (62.7)	142 (63.1)	108 (66.7)	103 (68.7)	57 (72.2)	52 (67.5)
Insufficient family support	149 (37.9)	165 (42.4)	113 (36.3)	119 (37.9)	91 (37.3)	83 (36.9)	54 (33.3)	47 (31.3)	22 (27.8)	25 (32.5)
Employed	19 (4.8)	24 (6.2)	6 (1.9)	11 (3.5)	3 (1.2)	2 (0.9)	1 (0.6)	0 (0.0)	0 (0.0)	0 (0.0
Not employed	374 (95.2)	365 (93.8)	305 (98.1)	303 (96.5)	241 (98.8)	223 (99.1)	161 (99.4)	150 (100.0)	79 (100.0)	77 (100.0)
Any arrest in prior six months[b]	28 (7.1)	19 (4.9)	3 (1.0)	3 (1.0)	0 (0.0)	0 (0.0)	0 (0.0)	0 (0.0)	0 (0.0)	0 (0.0)
Self-reported health: fair/good/excellent	182 (46.3)	181 (46.5)	144 (46.3)	145 (46.2)	110 (45.1)	94 (41.8)	68 (42.0)	63 (42.0)	34 (43.0)	33 (42.9)
Self-reported health: poor	211 (53.7)	208 (53.5)	167 (53.7)	169 (53.8)	134 (54.9)	131 (58.2)	94 (58.0)	87 (58.0)	45 (57.0)	44 (57.1)
Acquired immunodeficiency syndrome	12 (3.1)	4 (1.0)	9 (2.9)	3 (1.0)	6 (2.5)	2 (0.9)	2 (1.2)	1 (0.7)	0 (0.0)	1 (1.3)
Chronic obstructive pulmonary disease	180 (45.8)	168 (43.2)	158 (50.8)	147 (46.8)	135 (55.3)	114 (50.7)	99 (61.1)	87 (58.0)	58 (73.4)	48 (62.3)
Congestive heart failure	153 (38.9)	125 (32.1)	124 (39.9)	108 (34.4)	94 (38.5)	81 (36.0)	62 (38.3)	52 (34.7)	33 (41.8)	27 (35.1)
Dementia	6 (1.5)	2 (0.5)	6 (1.9)	1 (0.3)	5 (2.0)	1 (0.4)	2 (1.2)	0 (0.0)	1 (1.3)	0 (0.0)
Diabetes with complication	95 (24.2)	79 (20.3)	90 (28.9)	77 (24.5)	74 (30.3)	61 (27.1)	60 (37.0)	44 (29.3)	35 (44.3)	27 (35.1)
Hemiplegia or paraplegia	6 (1.5)	9 (2.3)	6 (1.9)	6 (1.9)	4 (1.6)	4 (1.8)	4 (2.5)	1 (0.7)	1 (1.3)	0 (0.0)
Mild liver disease	49 (12.5)	44 (11.3)	32 (10.3)	34 (10.8)	22 (9.0)	20 (8.9)	11 (6.8)	8 (5.3)	2 (2.5)	2 (2.6)

© 2023 Yang Q et al. *JAMA Network Open.*

eTable 1: Intervention and control group patient characteristics within increasingly distilled samples										
	**Percentage of population selected**									
	**100% (n=782)**		**80% (n=625)**		**60% (n=469)**		**40% (n=312)**		**20% (n=176)**	
	Treatment (n=393)	Control (n=389)	Treatment (n=311)	Control (n=314)	Treatment (n=244)	Control (n=225)	Treatment (n=162)	Control (n=150)	Treatment (n=79)	Control (n=77)
	**No. (%)**									
Moderate or severe liver disease	13 (3.3)	18 (4.6)	9 (2.9)	17 (5.4)	7 (2.9)	9 (4.0)	3 (1.9)	4 (2.7)	0 (0.0)	1 (1.3)
Renal disease	137 (34.9)	110 (28.3)	120 (38.6)	102 (32.5)	102 (41.8)	84 (37.3)	76 (46.9)	57 (38.0)	44 (55.7)	37 (48.1)
Rheumatoid arthritis	18 (4.6)	13 (3.3)	15 (4.8)	11 (3.5v	13 (5.3)	6 (2.7)	8 (4.9)	3 (2.0)	5 (6.3)	1 (1.3)
Alcohol use diagnosis	44 (11.2)	61 (15.7)	31 (10.0)	40 (12.7)	22 (9.0)	24 (10.7)	14 (8.6)	15 (10.0)	5 (6.3)	6 (7.8)
Substance use diagnosis	122 (31.0)	133 (34.2)	84 (27.0)	92 (29.3)	67 (27.5)	57 (25.3)	42 (25.9)	39 (26.0)	19 (24.1)	21 (27.3)
Anxiety disorder	87 (22.1)	88 (22.6)	72 (23.2)	59 (18.8)	52 (21.3)	41 (18.2)	33 (20.4)	30 (20.0)	18 (22.8)	18 (23.4)
Mood disorder	131 (33.3)	108 (27.8)	102 (32.8)	77 (24.5)	78 (32.0)	55 (24.4)	57 (35.2)	40 (26.7)	34 (43.0)	22 (28.6)
Schizophrenia	25 (6.4)	23 (5.9)	20 (6.4)	17 (5.4)	18 (7.4)	11 (4.9)	13 (8.0)	9 (6.0)	7 (8.9)	3 (3.9)
Suicide ideation	14 (3.6)	18 (4.6)	11 (3.5)	10 (3.2)	9 (3.7)	5 (2.2)	5 (3.1)	3 (2.0)	2 (2.5)	1 (1.3)

a.Number of hospitalizations in 6 months prior to trial enrollment, including the index admission during which the patient was enrolled in the trial.
b.Flags whether the patient had an arrest by the Camden County Police Department in the 6 months prior to trial enrollment.

© 2023 Yang Q et al. *JAMA Network Open.*

eFigure 2: Number of intervention hours received by treatment arm patients during their first week of intervention enrollment

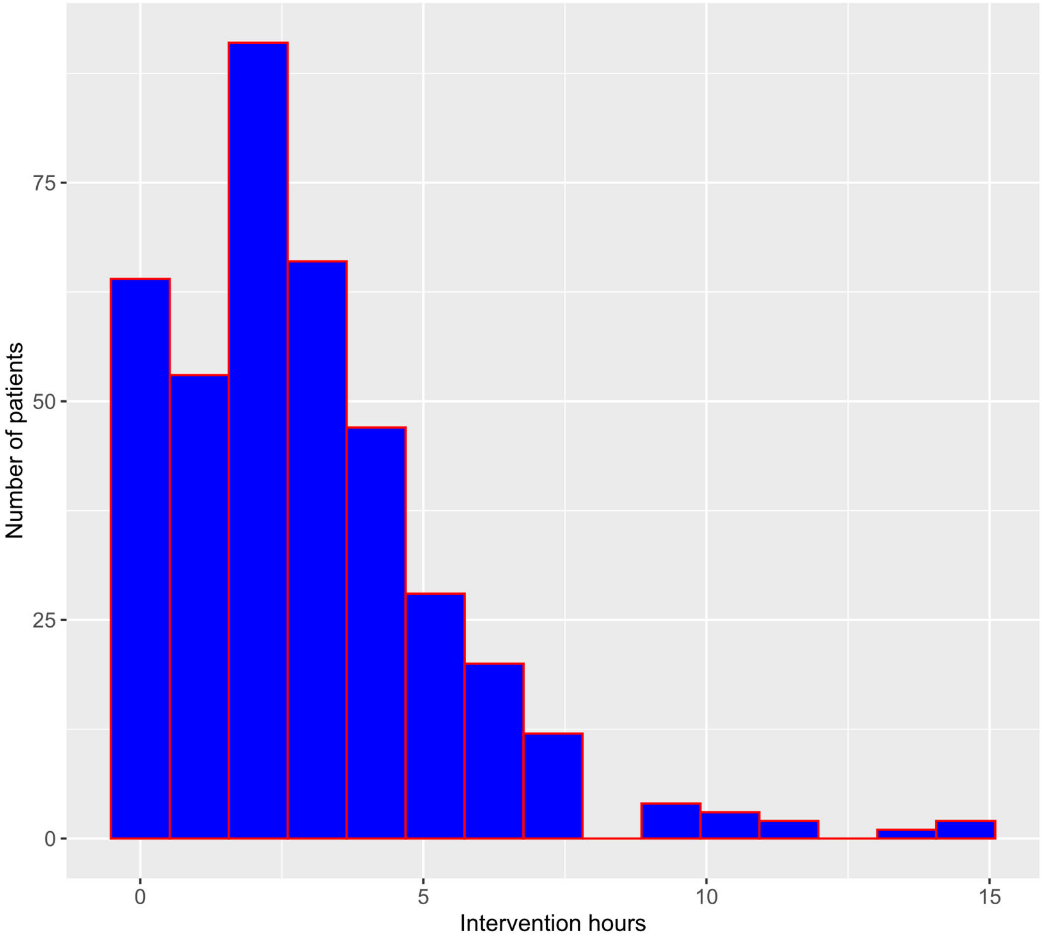

The data in the figure above cover 389 patients randomized to the intervention arm of the trial. Interactions that occurred between patients and care team staff either in person or on the phone were recorded in a care coordination database along with interaction length of time. The distribution ranges from 0 to 15 hours of engagement. Patients represented in the far-left bar received 1 hour or less of engagement during their first week of enrollment.

© 2023 Yang Q et al. *JAMA Network Open.*

eReferences

1. Finkelstein A, Zhou A, Taubman S, Doyle J. Health care hotspotting - a randomized, controlled trial. N Engl J Med. 2020 Jan 9;382(2):152-162.

2. Adams JL, Davis AC, Schneider EC, Hull MM, McGlynn EA. The distillation method: A novel approach for analyzing randomized trials when exposure to the intervention is diluted. Health Serv Res. 2022 Dec;57(6):1361-1369.

3. Quan H, Li B, Couris CM, et al. Updating and validating the Charlson comorbidity index and score for risk adjustment in hospital discharge abstracts using data from 6 countries. Am J Epidemiol. 2011;173(6):676-82.

4. Bozdogan, H. Model selection and Akaike's Information Criterion (AIC): The general theory and its analytical extensions. Psychometrika 52, 345–370 (1987).

5. Loann D, Review on Variable Selection in Regression Analysis. Econometrics 2018, 6(4), 45.

© 2023 Yang Q et al. *JAMA Network Open.*
