# Supplementary material for: Hospital Readmissions by Variation in Engagement in the Health Care Hotspotting Trial: A Secondary Analysis of a Randomized Clinical Trial
Source: JAMA Netw Open. 2023 Sep 12;6(9):e2332715. doi: 10.1001/jamanetworkopen.2023.32715 (PMC10498327; doi:10.1001/jamanetworkopen.2023.32715)
Supplement: Supplement 3. — Data Sharing Statement [file jamanetwopen-e2332715-s003.pdf]

Data Sharing Statement

Yang. Hospital Readmissions by Variation in Engagement in the Health Care Hotspotting Trial. *JAMA Netw Open*. Published September 12, 2023. doi:10.1001/jamanetworkopen.2023.32715

Data
Data available: Yes
Data types: Deidentified participant data
How to access data: https://dataverse.harvard.edu/dataset.xhtml?persistentId=doi:10.7910/DVN/ZJVVQZ
When available: beginning date: 06-06-2022

Supporting Documents
Document types: Statistical/analytic code
How to access documents: STATA syntax
When available: beginning date: 06-06-2022

Additional Information
Who can access the data: Anyone requesting data.
Types of analyses: Replication of the original clinical trial results.
Mechanisms of data availability: For access, data requestors must enter into a data access agreement with Harvard University
